# Unlocking circular bioeconomy potential of termite-gut yeasts: dual bioremediation and biodiesel production

**DOI:** 10.3389/fbioe.2025.1720952

**Published:** 2026-01-07

**Authors:** Sameh S. Ali, Min Xiong, Rania Al-Tohamy, Haixin Jiao, Michael Schagerl, Michael Kornaros, Jianzhong Sun

**Affiliations:** 1 Biofuels Institute, School of the Environment and Safety Engineering, Jiangsu University, Zhenjiang, China; 2 Botany Department, Faculty of Science, Tanta University, Tanta, Egypt; 3 School of Environmental Science and Engineering, Yancheng Institute of Technology, Yancheng, China; 4 Department of Functional and Evolutionary Ecology, University of Vienna, Vienna, Austria; 5 Laboratory of Biochemical Engineering and Environmental Technology (LBEET), Department of Chemical Engineering, University of Patras, Patras, Greece

**Keywords:** azo dyes, biodiesel production, bioremediation, circular bioeconomy, lignin-based aromatic wastes, microbial biorefineries, oleaginous yeasts, termite-gut yeasts

## Abstract

Lignin-derived aromatics and synthetic azo dyes are among the most persistent and toxic pollutants released by textile processing, petrochemical industries, pulp-and-paper manufacturing, and agricultural waste streams. Their structural complexity, chemical stability, and resistance to degradation impose substantial ecological and health concerns, highlighting the urgent need for sustainable and low-cost biological solutions. Growing evidence positions termite-gut symbioses—particularly yeast populations inhabiting wood-feeding termites—as a promising reservoir of biocatalysts capable of both degrading recalcitrant aromatic pollutants and generating lipids suitable for biodiesel production. This review synthesizes current knowledge on termite-gut-derived oleaginous yeasts, focusing on their enzymatic mechanisms, metabolic capabilities, and biotechnological potential within integrated biorefinery concepts. Recent literature reports demonstrate that termite-associated yeasts harbor diverse oxidative and reductive enzymes, including laccases, dye-decolorizing peroxidases, manganese peroxidases, dioxygenases, and azoreductases, which collectively mediate the depolymerization, detoxification, and mineralization of lignin-derived and dye-derived aromatic compounds. Pollutant-induced oxidative stress responses in oleaginous yeasts have also been widely documented to enhance lipid biosynthesis, linking environmental detoxification to biodiesel precursor generation through an energetically favorable, self-reinforcing metabolic cycle. Advances in genomics, transcriptomics, metabolic engineering, yeast surface display, and directed evolution have further expanded the opportunities to engineer multi-trait yeast chassis optimized for challenging industrial waste streams. This review also evaluates techno-environmental considerations relevant to practical deployment, including process scalability, tolerance to inhibitors, reactor configurations, and integration with lignocellulosic biorefineries and wastewater treatment systems. Particular attention is given to the potential of engineered termite-gut yeasts to function in hybrid microbial consortia, immobilized biocatalytic systems, and continuous-flow platforms. By consolidating the emerging scientific evidence, this review highlights termite-gut yeasts as a promising biological platform capable of bridging aromatic pollutant detoxification with renewable lipid production. Their dual functionality aligns strongly with circular bioeconomy goals, offering a path toward low-carbon, waste-to-value biorefineries.

## 1 Introduction

Aromatic pollutants, including lignin-derived aromatic polymers from agricultural residues and synthetic dyes from textile effluents, represent one of the largest and most problematic streams of renewable carbon sources (Nath et al., 2025). Their complex aromatic structures confer high chemical stability and recalcitrance, leading to persistence in soil and water systems and accumulation as emerging contaminants of global concern (Al-Tohamy et al., 2022). Polycyclic aromatic hydrocarbons (PAHs) and structurally related xenobiotics exemplify this challenge: their hydrophobicity reduces bioavailability, slows microbial degradation, and increases long-term ecological and human health risks (Sharma et al., 2025). Among these refractory aromatics, lignin-derived compounds and azo dyes dominate both natural and industrial waste streams. Lignin is one of the most abundant renewable aromatic biomaterials on Earth, constituting a significant portion of agro-industrial residues (Abdullah et al., 2023). In parallel, azo dyes—characterized by their azo (-N=N-) linkage to aromatic rings—are widely used in textiles due to their durability, colorfastness, and low cost (Emanuele and D’Auria, 2024). However, inefficient wastewater treatment and uncontrolled discharge from dye-intensive industries continue to introduce large quantities of these persistent pollutants into aquatic ecosystems. Given their structural similarity to lignin aromatics, azo dyes often require similar oxidative or reductive transformation pathways for effective remediation.

Bioremediation has emerged as an eco-efficient alternative to physical and chemical methods, enabling the conversion of toxic compounds into harmless metabolites through microbial enzymatic activity (Al-Tohamy et al., 2023a; Jiao et al., 2026). Enzyme systems such as laccases, lignin peroxidase (LiP), manganese peroxidase (MnP), and the recently identified dye-decolorizing peroxidases (DyPs) play critical roles in the oxidative breakdown of aromatic pollutants (Aragaw et al., 2024a; Kaur and Saini, 2024). However, their effectiveness is often limited by microbial robustness under stressful physicochemical conditions, such as salinity, pH variations, heavy metals, and inhibitory dye metabolites, which are frequently present in real wastewater matrices.

In addition to pollutant detoxification, increasing attention has been focused on the valorization of lignin-derived aromatics into renewable energy carriers, particularly microbial lipids for biodiesel production. Oleaginous yeasts have gained prominence as versatile cell factories capable of converting diverse carbon substrates—including aromatic monomers—into single-cell oils (SCOs) for sustainable biofuel generation (Bandhu et al., 2020). The dual functionality of pollutant degradation coupled with lipid biosynthesis presents a compelling strategy for integrating environmental remediation with waste-to-energy conversion. In this context, termite gut ecosystems represent one of nature’s most sophisticated lignocellulose-degrading bioreactors (Goux et al., 2023). Wood-feeding termites (WFTs) exhibit exceptional digestive efficiency, attributed to the synergistic actions of host enzymes and diverse gut symbionts (Scharf, 2020; Schwarz et al., 2023). Among these symbionts, yeasts isolated from termite guts have emerged as promising candidates for biotechnological applications due to their capacity to express ligninolytic and dye-transforming enzymes, as well as their ability to accumulate substantial intracellular lipids (Ali et al., 2020). Their natural tolerance to complex plant aromatics and inhibitory metabolites positions them as competitive alternatives to conventional oleaginous yeasts.

Microbial biorefinery concepts further strengthen the rationale for exploiting termite-gut yeasts. As reported by Al Azad et al. (2024), microbial biorefineries aim to integrate pollutant degradation, carbon recycling, and biofuel synthesis within unified, low-emission processes. Termite-derived yeasts—capable of both aromatic depolymerization and lipid accumulation—fit directly within this framework as emerging dual-function biocatalysts for circular bioprocesses. Despite growing interest in termite-gut yeasts, comprehensive comparisons with other microbial platforms remain limited. Key questions persist regarding their enzymatic advantages, stress tolerance, metabolic versatility, and scalability within integrated biorefinery systems. Addressing these gaps requires a synthesized evaluation of current knowledge spanning bioremediation enzymology, aromatic catabolism, and microbial lipid biosynthesis.

## 2 Lignin-based aromatic wastes

Lignin is one of the most abundant aromatic biopolymers on Earth and a major constituent of lignocellulosic biomass generated from forestry, agricultural, and agro-industrial activities. Together with cellulose and hemicellulose, lignin forms a complex lignocellulosic matrix in which carbohydrate polymers are tightly embedded within a phenolic network (Ali et al., 2026). This composite structure—stabilized by covalent linkages and hydrogen bonding—confers mechanical rigidity and natural resistance to chemical and biological attack (Huang et al., 2023). Chemically, lignin is a heterogeneous three-dimensional polymer synthesized from phenylpropanoid monomers interconnected by carbon–carbon and aryl–ether bonds. These structural attributes provide plants with physical protection and resilience but also render lignin highly recalcitrant to depolymerization (Yan et al., 2025). Its strong association with hemicellulose further complicates the release of fermentable sugars, posing a major challenge for biofuel and biochemical production.

Although lignin represents a vast renewable carbon reservoir—with global quantities estimated in the hundreds of billions of tons—its industrial utilization remains limited, and only a small fraction is commercially valorized (Kanwal et al., 2024). A large portion of lignin-rich residues arises from agricultural and forestry operations, making lignin-based biomass one of the most underexploited yet consistently available bioresources worldwide (Blasi et al., 2023). Inefficient management of these residues continues to pose environmental challenges: in many regions, open-field burning remains common, releasing particulate matter and toxic gases harmful to human health and contributing to greenhouse gas emissions and climate change (Kolawole and Iyiola, 2023). These concerns highlight the need for innovative strategies capable of depolymerizing and transforming lignin into value-added products rather than treating it as waste.

In parallel with natural lignin-derived aromatics, synthetic dyes—particularly azo dyes—contribute substantially to the global burden of aromatic pollutants. Azo dyes constitute the largest and most widely used class of synthetic dyes, accounting for a major share of the global dye market (Saini and Choudhary, 2025). Defined by their characteristic azo (–N=N–) linkage and aromatic ring structures, they are valued for their stability, vibrant coloration, and cost-effective production. Their extensive use spans textiles, leather, paper, pharmaceuticals, cosmetics, food processing, and printing technologies (Benkhaya et al., 2020). However, this widespread application is accompanied by significant losses to wastewater: global estimates suggest that 15% or more of produced dyes—equivalent to tens of thousands of tons annually—enter aquatic environments through effluent discharge, with textile wastewater often containing azo dye concentrations from trace levels up to >1,500 mg L^−1^ (Chequer et al., 2013; Affat, 2021). In regions with limited wastewater treatment infrastructure, direct or poorly treated discharge of dye-laden effluents remains a pervasive problem (Bhuvaneswari et al., 2020).

The environmental persistence and chromophoric stability of azo dyes make them among the most challenging industrial pollutants to remove. Their aromatic frameworks resist biodegradation, and reductive cleavage of azo bonds frequently generates aromatic amines that can be more hazardous than the parent compounds (Patel et al., 2023). These metabolites are associated with mutagenic, carcinogenic, and endocrine-disrupting effects and can accumulate in aquatic and terrestrial food webs (Ramamurthy et al., 2024). The intense coloration of dye-contaminated wastewater also restricts light penetration, suppressing photosynthesis in aquatic primary producers and altering ecosystem structure and function (Mehra et al., 2021). In agricultural settings, irrigation with dye-contaminated effluents has been shown to impair seed germination, root elongation, biomass accumulation, and chlorophyll content, reflecting combined osmotic stress and phytotoxicity of dyes and their breakdown products (Akter et al., 2023).

Toxicological and epidemiological studies further underscore the health risks associated with azo dye exposure. Laboratory investigations have reported oxidative stress, hepatotoxicity, nephrotoxicity, and genotoxic effects—including chromosomal aberrations and micronucleus formation—in animal models exposed to selected dyes and their metabolites (Tripathi et al., 2020; Ibitoye et al., 2022). Epidemiological data from textile dyeing and printing industries indicate elevated bladder cancer risk in workers exposed to certain benzidine-based azo dyes, consistent with metabolic activation of these compounds to carcinogenic aromatic amines (Sankhla et al., 2025). Collectively, these observations emphasize that both parent azo dyes and their reduction products can exert serious ecological and human health impacts.

The combined persistence, structural complexity, and toxicity of lignin-derived aromatics and azo dyes make them priority targets for sustainable remediation. Conventional physicochemical treatments often involve high operating costs and may generate secondary pollution (Al-Tohamy et al., 2022). By contrast, advances in microbial biotechnology—particularly the discovery of robust ligninolytic and azoreductase enzyme systems—offer promising avenues for converting these recalcitrant aromatic wastes into less toxic or value-added products (Rath et al., 2024). Integrating lignin depolymerization and dye detoxification with downstream bioprocesses such as microbial lipid production provides an opportunity to couple environmental remediation with the generation of biodiesel precursors, aligning with emerging microbial biorefinery concepts. Within this context, metabolically versatile microorganisms, including termite-gut-derived yeasts, are of particular interest because they can tolerate and transform structurally diverse aromatic pollutants while simultaneously accumulating lipids suitable for biofuel production.

## 3 Enzymatic toolbox for lignin- and dye-derived aromatic pollutants

**FIGURE 1:**
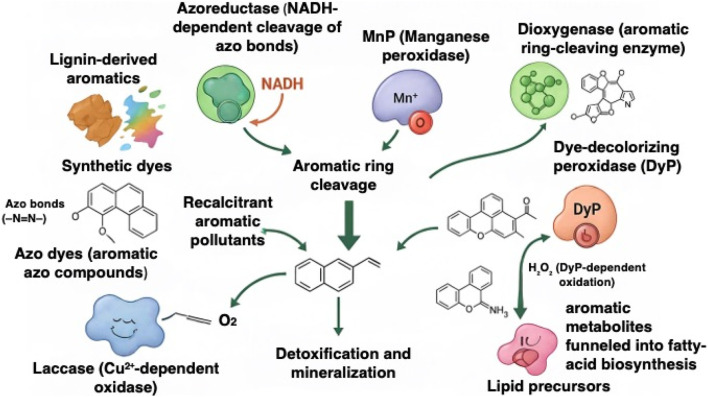
Enzymatic toolkit for microbial transformation of lignin- and dye-derived aromatic pollutants, where key oxidative and reductive enzymes collectively mediate the depolymerization, detoxification, and mineralization of recalcitrant aromatic compounds, enabling the conversion of lignin-derived monomers and dye fragments into less toxic or metabolically accessible intermediates.

The biodegradation and valorization of lignin-derived aromatics and synthetic dyes rely on a diverse suite of microbial enzymes capable of attacking highly recalcitrant aromatic structures. To illustrate the major enzymatic pathways implicated in the microbial breakdown of lignin-derived and dye-derived aromatic pollutants, Figure 1 summarizes the key oxidative and reductive enzymes involved in their transformation and detoxification. The most relevant classes include laccase, LiP, MnP, versatile peroxidases, and DyPs, which drive oxidative depolymerization, as well as azoreductases that mediate reductive cleavage of azo bonds (Kaur and Saini, 2024). White-rot fungi remain the classical model organisms for ligninolysis because of their powerful extracellular oxidoreductases. *Trametes versicolor* produces exceptionally high laccase activities (10^3^ U mL^−1^ range) and efficiently decolorizes dyes such as Indigo Carmine and RBBR under optimized acidic conditions (Ivanka et al., 2010). Members of the *Phanerochaete* genus, particularly *Phanerochaete sordida*, secrete combinations of laccase, LiP, and MnP that can decolorize and transform recalcitrant dyes like Reactive Black 5 under non-sterile conditions, demonstrating strong robustness in environmentally relevant matrices (Permpornsakul et al., 2016). These fungal systems set the benchmark for lignin and dye oxidation, although they often require controlled culture conditions and complex nutrient media.

**FIGURE 2:**
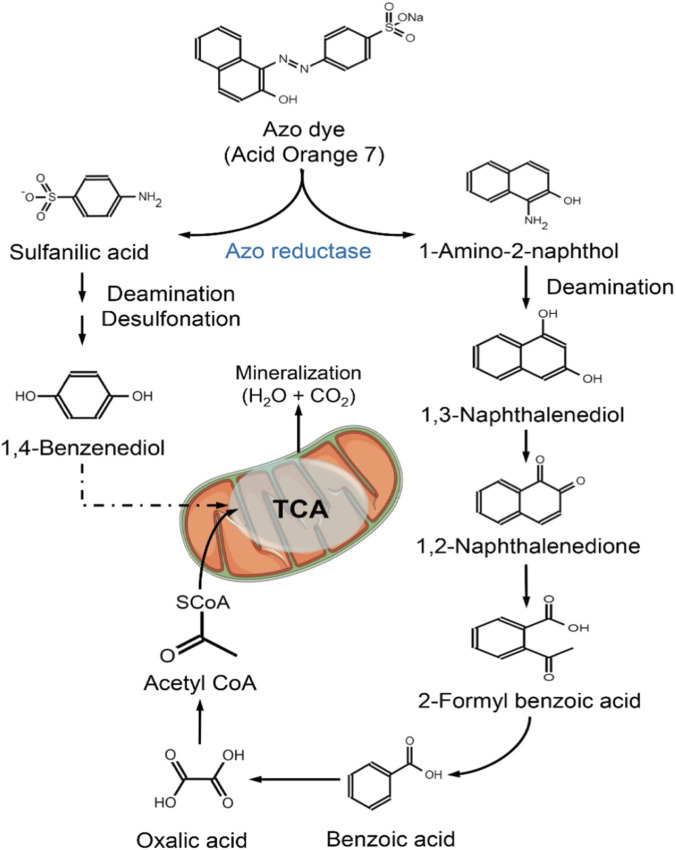
Biodegradation and mineralization pathway of Acid Orange 7 mediated by yeast azo reductase. This schematic illustrates the enzymatic cleavage of the azo dye Acid Orange 7 by yeast-derived azo reductase, generating two primary aromatic amines—sulfanilic acid and 1-amino-2-naphthol—as initial breakdown products. These intermediates undergo sequential deamination, desulfonation, and oxidation reactions to form compounds such as 1,4-benzenediol, 1,3-naphthalenediol, and 1,2-naphthalenedione. Further catabolic steps convert these molecules into low-molecular-weight organic acids, including benzoic acid and oxalic acid, which are subsequently funneled into the tricarboxylic acid (TCA) cycle as acetyl-CoA. This pathway culminates in complete mineralization to CO_2_ and H_2_O, demonstrating the capacity of yeast-based biocatalysis to achieve effective azo dye detoxification for environmental bioremediation.

In recent years, termite-gut-derived yeasts have emerged as promising alternatives combining ligninolytic activity with superior stress tolerance and simpler cultivation requirements. *Sterigmatomyces halophilus* SSA1575 expresses LiP and NADH–DCIP reductase and efficiently decolorizes RB5 across 50–1,500 mg L^−1^, even at NaCl concentrations up to 80 g L^−1^ (Al-Tohamy et al., 2020a). Likewise, the termite-derived MnP-producing oleaginous yeast consortium NYC-1, containing *Meyerozyma caribbica* SSA1654, achieves >98% decolorization of Acid Orange 7 (50 mg L^−1^) within 3 h and maintains high MnP activities (∼27 U mL^−1^) over broad pH and temperature ranges (Ali et al., 2021a). These performance metrics rival or exceed many fungal systems, especially under stressful conditions resembling real textile effluent environments. A distinguishing advantage of some termite-origin yeasts is their dual functionality: they simultaneously degrade aromatic pollutants and accumulate microbial lipids. *Meyerozyma caribbica* SSA1654, for instance, expresses high MnP activity during dye degradation while producing ∼47% lipid content (w/w CDW) with biodiesel-compatible C16–C18 fatty acids (Ali et al., 2021a). This contrasts with conventional oleaginous yeasts—e.g., *Yarrowia lipolytica*—which efficiently accumulate lipids on sugars and glycerol but lack strong native ligninolytic activity and therefore require pre-converted substrates (Sitepu et al., 2014; Dobrowolski et al., 2019). Reductive enzymes, particularly azoreductases, complement oxidative enzymes by cleaving azo bonds (–N=N–). Yeast azoreductases transform dyes such as Acid Orange 7 into aromatic amines that are subsequently processed through oxidative and ring-cleavage pathways before entering the TCA cycle (Al-Tohamy et al., 2020b). The pathway shown in Figure 2 illustrates this combined reductive–oxidative metabolism, leading to near-complete mineralization into CO_2_ and H_2_O.

Many catalytic metrics are obtained under optimized laboratory conditions that do not reflect industrial wastewater variability. Decolorization percentages, commonly used in dye studies, do not always correlate with detoxification or mineralization unless aromatic amines are measured (Ledakowicz and Paździor, 2021). Variability in reporting units, substrate type, and assay conditions also complicates comparisons across organisms. Therefore, standardized protocols—including both catalytic and ecotoxicological endpoints—are essential for benchmarking termite-gut yeasts against traditional fungal and bacterial platforms.

## 4 Major microbial enzymes for the utilization of lignin-based aromatics

The degradation and biotransformation of lignin-derived aromatics and synthetic dyes is fundamentally driven by specialized microbial enzymes capable of attacking highly stable aromatic structures. Understanding these enzymatic systems is essential not only for characterizing microbial biodegradation pathways but also for identifying robust candidates—such as termite-gut yeasts—capable of functioning in complex, inhibitor-rich environments relevant to integrated biorefineries. Table 1 provides a comparative context for these enzymatic mechanisms across different microbial systems.

**TABLE 1:** Microbial performance in aromatic dye degradation.

Microbial system	Key enzyme(s)	Model substrate(s)/Pollutant(s)	Performance metrics	References
*Trametes versicolor* (white-rot fungus)	Lac	Indigo Carmine, RBBR, phenolic dyes	• Crude Lac activities in the range of ∼1,000–2,000 U/mL • Complete decolorization of Indigo Carmine (0.01%) within a few hours at acidic pH and ∼40 °C–45 °C in optimized SSF systems, illustrating very high catalytic potential but requiring controlled conditions and often complex media	Ivanka et al. (2010)
*Phanerochaete sordida* PBU0057 (white-rot fungus)	Lac, LiP, MnP	RB5 (100 mg/L)	• >50% RB5 decolorization within 3 days for most isolates • Strain PBU0057 achieves ∼100% RB5 decolorization within 3 days under non-sterile, environmentally relevant conditions, supported by measurable LiP/MnP activities • Demonstrates robustness but relatively slow kinetics compared with high-performance yeast systems	Permpornsakul et al. (2016)
*Sterigmatomyces halophilus* SSA1575 (termite-gut-derived yeast)	LiP, NADH-DCIP reductase, other oxidoreductases	RB5 (50–1,500 mg/L), mixed azo dyes, high salinity	• Efficient decolorization of RB5 up to 1,500 mg/L • Maintains high decolorization at NaCl up to 80 g/L • Multiple dye additions decolorized within ≤18–24 h. Identified LiP and NADH-DCIP reductase as key activities • Highlights exceptional halotolerance and suitability for saline textile effluents	Al-Tohamy et al. (2020b)
MnP-producing oleaginous yeast consortium NYC-1 (termite-gut-derived yeasts, including *Meyerozyma caribbica* SSA1654)	MnP, Lac, azoreductases, auxiliary oxidoreductases	AO7 (50–250 mg/L)	• >98% decolorization of 50 mg/L AO7 within 3 h • >92% decolorization at 250 mg/L within 18 h • MnP activities up to ∼27 U/mL • Effective over broad pH (5–9), temperature (28 °C–50 °C) and moderate salinity, showing high kinetic efficiency and stress tolerance	Al-Tohamy et al. (2021)

Abbreviations: Lac, Laccase; LiP, lignin peroxidase; MnP, manganese peroxidase; NADH-DCIP, reductase, Nicotinamide adenine dinucleotide– 2,6-dichlorophenol-indophenol reductase; RB5, Reactive Black 5; AO7, Acid Orange 7; RBBR, Remazol Brilliant Blue R.

### 4.1 Key oxidative enzymes in lignin degradation

The biological conversion of lignin-derived aromatics relies predominantly on oxidative enzymes that activate and cleave stable aromatic structures, thereby generating intermediates that can be funneled into central metabolism or further transformed into value-added products. Among these, oxygenases, laccase, LiP, MnP, versatile peroxidase (VP), and related high-redox-potential oxidoreductases form the core catalytic toolbox. Comprehensive reviews on lignin biodegradation emphasize that these enzymes, acting alone or in concert, mediate the initial oxidative attack on phenolic and non-phenolic lignin units and on structurally related xenobiotic aromatics (Wang et al., 2018; Cagide and Castro-Sowinski, 2020; Singh et al., 2024). Oxygenases, primarily monooxygenases and dioxygenases, catalyze the incorporation of one or two oxygen atoms into aromatic substrates, typically using NADH or NADPH as electron donors. This reaction destabilizes aromatic rings, increases hydrophilicity, and facilitates subsequent ring-cleavage and mineralization steps. They play key roles in the microbial degradation of polycyclic aromatic hydrocarbons, chlorophenols, and other persistent pollutants, especially in bacteria such as *Pseudomonas*, *Sphingomonas*, and *Burkholderia*, but also in selected fungi and yeasts (Saravanan et al., 2021). These enzymes establish the upstream routes that connect complex lignin-derived fragments with central catabolic pathways.

Laccases (EC 1.10.3.2), multicopper oxidases widespread in fungi, bacteria, plants, and some insects, catalyze the one-electron oxidation of a wide range of phenolic and certain non-phenolic substrates while reducing O_2_ to water. Their broad substrate scope and ability to act with redox mediators make them central to lignin depolymerization, dye decolorization, and phenolic pollutant transformation (Janusz et al., 2020). However, many fungal laccases operate optimally under mildly acidic conditions and show sensitivity to halides and inhibitors, which can limit their performance in saline, alkaline, or heavily contaminated industrial effluents (Abdi Dezfouli and Esmaeilidezfouli, 2024). This constraint has motivated the search for more robust laccase variants and non-conventional producers. Among the most powerful oxidative enzymes in white-rot fungi, the heme-containing peroxidases LiP and MnP possess sufficiently high redox potentials to attack recalcitrant aromatic structures that lie beyond the catalytic reach of common oxidases (Baker et al., 2019). LiP, using H_2_O_2_ and mediators such as veratryl alcohol, oxidizes non-phenolic lignin units and various aromatic pollutants; MnP oxidizes Mn^2+^ to Mn^3+^, forming diffusible Mn^3+^–organic acid complexes that attack phenolic structures within lignocellulosic matrices (Biko et al., 2020). Together, they play a decisive role in lignin fragmentation and in the transformation of xenobiotic aromatics embedded in complex substrates.

Versatile peroxidase (VP; EC 1.11.1.16) combines catalytic features of both LiP and MnP, being able to oxidize Mn^2+^, phenolic substrates, and non-phenolic lignin model compounds without requiring specific mediators, thus broadening its substrate range. Recent structure–function and engineering studies have highlighted VP as a promising biocatalyst for lignin valorization and pollutant degradation due to its high redox potential and flexible active site architecture (Barber-Zucker et al., 2022; Singh et al., 2024). Nonetheless, many of these classical peroxidases and laccases display limited stability at extreme pH, elevated temperatures, high salinity, or in the presence of industrial inhibitors (e.g., dyes, lignocellulose-derived aldehydes), which restricts their direct application in harsh real-world waste streams. This limitation underscores the importance of identifying naturally robust enzyme systems—such as those emerging from termite-gut-derived yeasts—that retain ligninolytic activity under inhibitory and fluctuating conditions relevant to integrated biorefineries.

### 4.2 Distinctive ligninolytic systems of termite-gut-derived yeasts

The digestive system of wood-feeding termites represents a natural high-performance lignocellulose bioreactor, where complex plant polymers are efficiently deconstructed under microaerophilic conditions and in the presence of lignin-derived aromatics, organic acids, and inorganic ions (Dumond et al., 2021). Within this niche, yeasts form part of a specialized symbiotic community that has undergone strong selection for oxidative and reductive capacities compatible with lignin-rich and inhibitor-laden substrates (Ali et al., 2018). Recent work has demonstrated that termite-gut-derived yeasts frequently co-express MnP, LiP- or LiP-like activities, laccase, azoreductase, NADH–DCIP reductase, and glycosidases, generating a highly synergistic enzymatic repertoire capable of transforming structurally diverse aromatic pollutants while supporting growth and, in several cases, lipid accumulation (Al-Tohamy et al., 2020a; Ali et al., 2021a).

A key example is the MnP-producing oleaginous yeast *M. caribbica* SSA1654, isolated from wood-feeding termite gut symbionts. Screening studies on termite-gut yeast collections identified multiple isolates with high extracellular MnP activity, efficient azo dye decolorization, and oleaginous phenotypes; *M. caribbica* SSA1654 was among the best performers, achieving rapid decolorization of sulfonated azo dyes such as Acid Orange 7 over a broad concentration range while producing lipids with fatty acid profiles suitable for biodiesel (Ali et al., 2022). These results demonstrate that ligninolytic activity and lipid biosynthesis can coexist in a single termite-derived yeast, directly supporting a dual-function concept where pollutant removal and biofuel precursor generation occur simultaneously. Further mechanistic work on *M. caribbica* SSA1654 has shown that its MnP activity and dye degradation performance are maintained in the presence of lignocellulosic inhibitors, and that the lipids produced under dye or inhibitor exposure retain physicochemical properties compatible with international biodiesel standards (Al-Tohamy et al., 2021). This robustness contrasts with many conventional fungal or yeast ligninolytic systems, which are often inhibited by similar stressors, and highlights termite-gut yeasts as sources of enzyme systems adapted to chemically complex environments.

Termite-associated halotolerant yeasts provide additional evidence for distinctive ligninolytic capacities. *Sterigmatomyces halophilus* SSA1575, isolated from termite gut, has been reported to efficiently decolorize Reactive Black 5 and other azo dyes under high salinity and variable physicochemical conditions, with detoxification confirmed by phytotoxicity and ecotoxicity assays (Al-Tohamy et al., 2020b). This strain expresses both MnP and reductive enzymes such as NADH–DCIP reductase, enabling complementary oxidative and reductive cleavage of dye structures in saline textile wastewater. More recently, a halotolerant yeast consortium HYC derived from termite-associated strains, dominated by *S. halophilus* SSA1575 and *Meyerozyma guilliermondii* SSA1547, demonstrated high decolorization efficiencies of Reactive Black 5 at elevated dye concentrations and NaCl levels up to 50 g L^−1^, further confirming the resilience and cooperative ligninolytic potential of termite-derived yeasts under conditions that limit many classical fungal systems (Al-Tohamy et al., 2023b).

Collectively, these studies indicate that termite-gut-derived yeasts possess ligninolytic systems with several distinguishing features compared with conventional lignin-degrading microbes. First, they exhibit multi-enzyme synergy, in which MnP, laccase-like activities, azoreductases, and accessory oxidoreductases function together within the same strain or consortium to attack a wide spectrum of aromatic substrates, including lignin-derived phenolics and synthetic dyes (Al-Tohamy et al., 2021). Second, they display enhanced tolerance to conditions relevant to industrial effluents—such as moderate salinity, variable pH, and the presence of inhibitory lignocellulose-derived compounds—while maintaining high decolorization or degradation efficiencies (Al-Tohamy et al., 2020a). Third, in strains such as *M. caribbica* SSA1654 and termite-derived MnP-producing consortia, ligninolytic activity is coupled to oleaginous metabolism, enabling the conversion of aromatic pollutants into intracellular lipids that can be upgraded to biodiesel, thereby integrating bioremediation with bioenergy production (Al-Tohamy et al., 2021). This dual-functionality closely aligns with the broader microbial biorefinery paradigm, in which pollutant degradation and biofuel synthesis are unified within a single, resource-efficient biological framework. Recent analyses by Al Azad et al. (2024) have emphasized that such integrated systems—linking lignin depolymerization, carbon recycling, and lipid generation—represent a cornerstone of circular bioeconomy strategies aimed at reducing both carbon footprint and process waste. These properties position termite-gut-derived yeasts not merely as alternative hosts for known ligninolytic enzymes but as evolutionarily selected, functionally integrated ligninolytic–oleaginous systems. Their enzyme portfolios and stress-tolerant phenotypes provide a mechanistic foundation for the sustainable utilization of lignin-based aromatics in single-platform biorefineries, justifying their central role in the present review and aligning directly with the manuscript’s focus on termite-gut-based integrated bioprocesses.

Despite significant progress in elucidating ligninolytic enzymes and microbial pathways, numerous methodological limitations persist across reported studies. Laboratory assays often employ model compounds (e.g., guaiacol, vanillic acid) or simplified lignin analogues that differ markedly from industrial lignin and field-derived aromatic pollutants, leading to overestimation of degradation efficiency (Janusz et al., 2017). Moreover, enzyme kinetics are commonly determined under controlled pH and temperature conditions that fail to capture the physicochemical variability of real effluents, where inhibitory metals, surfactants, and fluctuating redox states markedly reduce catalytic turnover (Atiwesh et al., 2022). Scaling from flasks to bioreactors introduces additional challenges, including oxygen transfer limitations, shear sensitivity, and unstable cofactor regeneration, which can suppress the activity of peroxidases and oxygenases by more than an order of magnitude (Kumar and Chandra, 2020). Another critical issue involves inconsistent reporting of enzyme units and lack of standardized substrates, hindering cross-comparison of results among research groups (Atiwesh et al., 2022). Addressing these methodological gaps through harmonised testing protocols, realistic lignin matrices, and continuous bioprocess evaluation will be essential to translate current enzymatic insights into scalable, environmentally relevant applications.

### 4.3 Yeasts as promising candidates for the bioremediation of lignin-derived aromatics

Yeasts have emerged as powerful biocatalysts for the remediation of lignin-derived aromatics and synthetic textile dyes due to their unique physiological and biochemical advantages compared with bacteria and filamentous fungi. Their rapid growth, high tolerance to physicochemical stress, and capacity to function under acidic pH, variable salinity, and high pollutant loads make them particularly suitable for industrial wastewater treatment settings (Calabon et al., 2023; Velasquez-Orta and Mohiuddin, 2023). Unlike filamentous fungi—which often suffer from slow biomass formation and morphological instability—yeasts maintain robust performance in continuous or semi-continuous systems and demonstrate resilience in effluents containing salts, surfactants, heavy metals, and complex aromatic mixtures (Wen et al., 2022; Mohiuddin et al., 2024). Yeasts employ a combination of biosorption, bioaccumulation, and enzymatic biodegradation to transform and detoxify aromatic pollutants. Biosorption—driven by ion exchange, complexation, and electrostatic interactions between dye molecules and cell wall polymers—represents a rapid, non-metabolic removal mechanism that is particularly effective in high-strength effluents (Gouda and Taha, 2023). Bioaccumulation, in contrast, involves active intracellular uptake and metabolic conversion, allowing cells to transform xenobiotics into less toxic intermediates (Ayele and Godeto, 2021). These two pathways frequently act synergistically, with biosorption providing an immediate reduction in pollutant concentrations while bioaccumulation and enzymatic degradation enable complete detoxification.

From a biochemical standpoint, the strength of yeast-based systems lies in their diverse oxidoreductase repertoire, including laccases, peroxidases, tyrosinases, azoreductases, and NADH–DCIP reductases (Al-Tohamy et al., 2020a; Ruscasso et al., 2022). These enzymes collectively target phenolic and non-phenolic aromatics, azo bonds, and various dye chromophores. Laccases offer broad substrate flexibility and can function with redox mediators, whereas tyrosinases are effective in the oxidation of monophenolic and diphenolic contaminants (Rane and Joshi, 2021; de Mello et al., 2023). Reductive enzymes—particularly azoreductase and NADH–DCIP reductase—initiate cleavage of azo bonds and play a key role in maintaining intracellular redox balance during pollutant stress (Aragaw, 2024). The upregulation of reductive enzymes observed during dye treatment further illustrates yeast metabolic adaptability to toxic xenobiotics (Danouche et al., 2021).

Despite these advantages, several constraints limit industrial deployment of yeast-based bioremediation. Many studies rely on model dyes or lignin analogues tested under optimized laboratory conditions, which do not reflect the complexity of real industrial effluents containing high salinity, variable pH, heavy metals, and recalcitrant aromatic mixtures (Janusz et al., 2020; Al-Gethami et al., 2024). Furthermore, enzyme activities and pollutant removal rates often decrease significantly upon scaling due to oxygen transfer limitations, shear stress, nutrient gradients, and cofactor regeneration challenges that inhibit peroxidases and oxygenases (Khaliq, 2023). Techno-economic analyses indicate that aeration, mixing, and biomass harvesting are the primary cost drivers in continuous reactor designs, often exceeding 50% of total operating expenses, while pollutant removal efficiencies in continuous systems can drop by 30%–50% compared with batch operations (Bala et al., 2022; Mohiuddin et al., 2024).

A major opportunity lies in integrating pollutant degradation with metabolic valorization, such as lipid accumulation and conversion to biodiesel. However, achieving industrial feasibility requires overcoming bottlenecks associated with energy demand, lipid extraction costs, and process throughput. Economic viability improves when lipid titers exceed ∼5 g L^−1^ and remediation throughput reaches ≥1 m^3^ h^−1^ (Mohiuddin et al., 2024). To address these challenges, future research must incorporate multi-omics, metabolic engineering, and bioprocess modeling, ensuring robust enzyme expression, improved stress tolerance, and optimized redox metabolism. Integrating yeast-driven detoxification within circular biorefinery frameworks—as emphasized by Al Azad et al. (2024)—may reduce resource input, valorize waste-derived carbon, and enhance overall sustainability. Collectively, yeasts offer a strong foundation for aromatic pollutant remediation due to their robustness, metabolic flexibility, and ability to couple detoxification with bioresource generation. Yet their successful scale-up requires harmonized testing with real effluents, integrated techno-economic analysis (TEA) and life-cycle assessment (LCA) assessments, and strategic engineering interventions to bridge the persistent gap between laboratory promise and industrial feasibility.

## 5 Termite-gut yeasts as dual bioremediation–biodiesel platforms

Building upon the enzymatic foundations outlined in Section 3, it is increasingly evident that termite-gut-derived yeasts offer far more than isolated ligninolytic activities. These symbiotic yeasts integrate robust oxidative and reductive enzymes with whole-cell metabolic traits that enable simultaneous pollutant degradation and lipid biosynthesis. Therefore, this section examines termite-gut yeasts as complete dual-function platforms—capable of coupling bioremediation of lignin-derived aromatics and dyes with the production of biodiesel-compatible lipids—positioning them as emerging cornerstones in next-generation microbial biorefineries.

### 5.1 Diversity and ecological origin of termite-gut yeasts

The gut of wood-feeding termites is recognized as a highly specialized lignocellulose-degrading bioreactor in which host-derived enzymes and a dense, diverse microbiota act in concert to deconstruct plant biomass under tightly structured physicochemical gradients (Brune, 2014; Xie et al., 2024). While bacteria and protozoa dominate many functional niches, culture-based and molecular studies have confirmed that yeasts are recurring members of termite gut communities and contribute to carbohydrate turnover, nutrient supply, and detoxification of plant-derived compounds (Tiwari et al., 2020). Surveys of wood-feeding termite guts and other insect digestive systems have revealed a broad yeast diversity dominated by ascomycetous taxa, including *Candida*, *Debaryomyces*, *Meyerozyma*, *Pichia*, and related genera (Tiwari et al., 2020). Recent high-throughput analyses of termite-associated microbial assemblages have specifically highlighted *Debaryomyces* and *Meyerozyma* as typical and abundant gut inhabitants in certain termite hosts, suggesting a stable association rather than transient colonization (Chakraborty et al., 2023). Parallel work on insect-associated yeasts more broadly indicates that such symbionts often support the host by contributing vitamins, enzymes for polymer degradation, and the detoxification of allelochemicals, while benefiting in turn from a protected, nutrient-rich niche (Malassigné et al., 2021).

Several ecological pressures within termite hindguts likely drive the evolution of the distinctive traits now observed in termite-derived yeasts. First, the diet of wood-feeding termites is enriched in lignocellulosic material containing lignin-derived aromatics and phenolic inhibitors, which means resident yeasts must tolerate and often transform a range of aromatic compounds to survive and grow (Brune, 2014). Second, the hindgut environment is microaerophilic and strongly stratified in redox potential, with steep oxygen gradients and fluctuating concentrations of fermentation products such as acetate, hydrogen, and short-chain organic acids; these conditions favor facultative and metabolically flexible yeasts capable of switching between oxidative and fermentative modes (Brune, 2014). Third, the continual influx of partially degraded plant polymers and mineral ions imposes osmotic and ionic stresses, under which osmotolerant or halotolerant yeasts gain a selective advantage (Tiwari et al., 2020).

These ecological and evolutionary drivers are consistent with the phenotypes reported for termite-gut-derived yeasts in recent biotechnological studies. Screening of yeasts isolated from wood-feeding termite gut symbionts has revealed manganese peroxidase–producing oleaginous strains, notably *M. caribbica* SSA1654 and related isolates, that combine growth in the presence of lignocellulose-derived inhibitors (furfural, 5-hydroxymethylfurfural, acetic acid, vanillin, formic acid) with substantial intracellular lipid accumulation and the ability to transform recalcitrant azo dyes (Ali et al., 2021a). Complementary work has identified termite-gut symbionts as a reservoir of yeasts with xylanolytic and ethanologenic potential, underlining that this niche selects for strains capable of utilizing complex polysaccharides and mixed sugar streams derived from lignocellulose. A broader state-of-the-art review has further emphasized that yeasts from termite guts constitute a largely untapped resource with combined capacities for lignin- and dye-derived aromatic valorization and lipid-based biofuel production (Ali et al., 2022).

Together, these findings support the view that termite-gut yeasts are not incidental colonizers but ecologically specialized symbionts shaped by chronic exposure to lignocellulosic substrates, lignin-derived inhibitors, fluctuating redox conditions, and nutrient limitation. The same adaptations that enable them to endure and process aromatic-rich diets in situ—tolerance to inhibitors, flexible carbon utilization, and propensity for lipid storage—also make them attractive as dual-function biocatalysts for bioremediation of aromatic wastes and biodiesel-oriented microbial biorefineries, justifying their central role in the subsequent sections of this review.

### 5.2 Ligninolytic and dye-degrading performance of termite-gut yeasts

Termite-gut-derived yeasts have attracted growing attention because they can sustain high ligninolytic and dye-degrading activities under conditions that mimic real textile and lignocellulosic effluents. A key example is the halotolerant strain *S. halophilus* SSA-1575, which was originally isolated from wood-feeding termite guts. This yeast was shown to decolorize a wide range of sulfonated azo dyes, including Reactive Black 5, with removal efficiencies often exceeding 90% across dye concentrations up to 1,500 mg L^−1^ and NaCl levels up to 80 g L^−1^ under optimized conditions (Al-Tohamy et al., 2020b). In addition to rapid decolorization, phytotoxicity and ecotoxicity assays demonstrated substantial detoxification of treated effluents, indicating that *S. halophilus* SSA-1575 converts highly colored and toxic dye mixtures into products with significantly reduced environmental impact (Al-Tohamy et al., 2020b). A complementary study confirmed its performance in continuous and high-salt regimes, further supporting its suitability for saline textile wastewater treatment (Al-Tohamy et al., 2020a).

Beyond single strains, termite-derived yeast consortia display even higher kinetic performance and robustness. A MnP-producing oleaginous yeast consortium NYC-1, enriched from termite-gut isolates and dominated by *Meyerozyma* and *Sterigmatomyces* spp., achieved >98% decolorization of Acid Orange 7 (50 mg L^−1^) within 3 h and >92% decolorization at 250 mg L^−1^ within 18 h, while maintaining activity across a broad pH (5–9), temperature (28 °C–50 °C), and moderate salinity window (Al-Tohamy et al., 2021). Importantly, these studies used model dyes supplemented with lignocellulosic residues and common lignin-derived inhibitors (e.g., vanillin, furfural), showing that the consortium retains high decolorization efficiency even in the presence of compounds that typically suppress fungal peroxidases and laccases. At the single-strain level, *M. caribbica* SSA1654 illustrates how termite-gut yeasts combine rapid dye degradation with tolerance to aromatic inhibitors. This strain, identified as a novel MnP producer inhabiting wood-feeding termite gut symbionts, decolorized sulfonated azo dyes such as Acid Orange 7 across a wide concentration range under mildly saline conditions and in the presence of lignocellulose-derived inhibitors (Ali et al., 2022). Such performance is comparable to, or better than, many classical white-rot fungi operating in simpler matrices, while offering the added advantages of unicellular growth and easier process control.

When benchmarked against salt-tolerant non-termite yeasts, termite-derived strains perform competitively or better under high-salt and high-dye conditions. For example, *Pichia occidentalis* G1 decolorized a variety of azo dyes with >98% removal for several substrates under saline conditions (up to 70 g L^−1^ NaCl) (Song et al., 2017). Similarly, *Scheffersomyces spartinae* TLHS-SF1 and *Cyberlindnera samutprakarnensis* S4 achieved >90–97% decolorization of Acid Red B and other azo dyes under high-salt regimes (Tan et al., 2016). However, these systems are primarily designed for decolorization and detoxification and are not inherently oleaginous. In contrast, termite-derived yeasts and consortia frequently couple high dye-degrading activity with lipid accumulation and tolerance to lignin-like substrates, offering additional value for integrated biorefinery applications (Ali et al., 2025).

Overall, the available data show that termite-gut yeasts and their consortia can match or exceed the decolorization kinetics of established salt-tolerant yeast platforms while operating efficiently in more chemically complex matrices that contain both textile dyes and lignin-derived aromatics. Their ability to maintain high performance under inhibitory, saline, and variable pH conditions supports their positioning as promising core biocatalysts for dual-function systems that combine bioremediation of aromatic pollutants with downstream lipid valorization.

### 5.3 Oleaginous metabolism and biodiesel properties

The same termite-gut-derived yeasts that excel in dye degradation also display strong oleaginous phenotypes, enabling direct coupling of pollutant removal with SCO production. *Meyerozyma caribbica* SSA1654, for instance, accumulates lipids up to ∼47% of its DCW when grown on glucose-based media while simultaneously expressing high MnP activity and decolorizing sulfonated azo dyes (Ali et al., 2022). The fatty acid profile of SSA1654 lipids is dominated by C16–C18 species, with a balance of saturated and monounsaturated chains that yields predicted biodiesel properties—cetane number, kinematic viscosity, cold filter plugging point, and oxidative stability—within or close to EN 14214 limits (Ali et al., 2022). Notably, exposure to dyes and lignocellulosic inhibitors does not drastically impair lipid yield or quality, indicating that this strain can convert stress-inducing aromatic substrates into biodiesel-grade lipids under realistic effluent conditions (Lyu et al., 2025).

At the consortium level, NYC-1, composed of MnP-producing termite-derived yeasts, exemplifies integrated bioremediation–biodiesel functionality. During Acid Orange 7 degradation, NYC-1 not only achieves rapid decolorization but also accumulates lipids with total contents often exceeding 40% of biomass, producing a FAME mixture enriched in C16:0, C18:0, C18:1, and C18:2 (Al-Tohamy et al., 2021). Fuel property modeling showed that the resulting biodiesel has a cetane number around 53 and oxidative stability and viscosity within EN 14214 standards (Kivevele and Huan, 2013), confirming that azo dye-derived carbon can be recycled into transportation-grade fuel precursors without sacrificing fuel quality.

Termite-derived yeasts also compare favorably with non-termite oleaginous yeast consortia developed for dyeing wastewater and lignin-like dye valorization. A multipurpose oleaginous yeast consortium (OYC-Y.BC.SH), constructed from *Yarrowia* sp. SSA1642, *Barnettozyma californica* SSA1518, and *S. halophilus* SSA1511, reached lipid productivities of ∼1.56 g L^−1^ day^−1^ while decolorizing textile dyes and real dyeing effluents, with FAME profiles suitable for biodiesel production (Ali et al., 2021b). A related cold-adapted consortium, constructed for textile azo dye wastewater processing, also produced biodiesel-compatible lipids at low operating temperatures, illustrating the feasibility of tailoring yeast consortia for specific climatic and effluent conditions. Termite-derived lineages contribute key members and enzymatic traits to these consortia, particularly high MnP output and tolerance to lignin-like dyes, strengthening their role in integrated bioprocesses.

When viewed in the broader context of oleaginous yeast biotechnology, termite-gut yeasts occupy a distinctive niche. Many conventional oleaginous yeasts, such as *Cryptococcus curvatus*, *Rhodotorula*/*Rhodosporidium* spp., and *Y. lipolytica*, achieve lipid contents of 40%–70% DCW on sugars, glycerol, or simple waste substrates, but often require pre-detoxified feedstocks with low levels of aromatic inhibitors (Broos et al., 2022). Recent reviews on biodiesel production from oleaginous yeasts emphasize that valorization of recalcitrant wastes, such as lignin-rich residues and dyeing effluents, is promising but still constrained by microbial stress and low conversion efficiency (Sartaj et al., 2023). In this setting, termite-derived yeasts and consortia stand out because they were selected *in vivo* for survival in lignin-rich, aromatic, and inhibitor-laden environments, and thus naturally integrate ligninolytic activity with robust lipid biosynthesis.

From a circular bioeconomy perspective, the capacity of termite-gut yeasts to convert lignin-derived aromatics and textile dyes into biodiesel-grade lipids offers an elegant route to close the carbon loop: the same aromatic structures that cause recalcitrant pollution are repurposed as feedstocks for renewable fuels. However, as highlighted for yeast-based systems in general, full techno-economic and life-cycle assessments are still needed to determine whether lipid titers, volumetric productivities, and energy demands can meet industrial thresholds when real effluents are used at scale (Broos et al., 2022; Sartaj et al., 2023). In this context, termite-gut-derived yeasts provide a strong biological starting point—combining dye degradation, lignin valorization, and SCO formation—but their performance must be further optimized through strain engineering, adaptive evolution, and process integration with upstream pretreatment and downstream lipid recovery. To contextualize termite-gut yeasts within broader ligninolytic and oleaginous microbial platforms, Table 2 summarizes representative organisms exhibiting combined (or complementary) capacities for lignin modification, dye degradation, and single-cell oil production.

**TABLE 2:** Lignin-modifying enzyme producers for the bioremediation of model lignin compounds and industrial dyes.

Microbial system	Substrate(s)/Effluent	Coupled functions	Performance metrics	References
Engineered *Yarrowia lipolytica* AJD pAD-DGA1	Crude glycerol (150 g/L), seawater-based medium	Waste valorization to SCO	• Lipid contents up to ∼37–43% CDW in shake flasks • ∼10 g/L lipids in 5 L bioreactor using crude glycerol + seawater • Demonstrates robust SCO production from low-cost/waste substrates but without intrinsic ligninolytic activity, requiring pretreated feeds	Dobrowolski et al. (2019)
*Sterigmatomyces halophilus* SSA1575	RB5 (up to 1,500 mg/L), mixed dyes, high-salt textile wastewater	Halotolerant azo dye degradation + detoxification (no lipid focus)	• Near-complete RB5 decolorization even at very high dye loadings and NaCl up to 80 g/L • Confirmed reduction in phytotoxicity and ecotoxicity of treated effluents, illustrating suitability for saline and recalcitrant waste streams	Al-Tohamy et al. (2020b)
Oleaginous yeasts (general, conventional platforms)	Sugars, crude glycerol, agro-residues	SCO/biodiesel precursors (no direct lignin depolymerization)	• Numerous strains reach 20%–70% lipid of CDW on optimized substrates • Most lack strong native ligninolytic capacity and require pre-converted streams, contrasting with termite-derived yeasts that combine pollutant degradation and lipid synthesis in a single system	Sitepu et al. (2014)
*Meyerozyma caribbica* SSA1654	AO7 and related azo dyes; glucose-based media	MnP-mediated dye degradation + single-cell oil production	• High MnP activity associated with rapid dye decolorization • Lipid content 47.25% ± 1.84% (w/w) of CDW with C16–C18 profile; predicted biodiesel properties (cetane number, kinematic viscosity, oxidative stability) meet EN 14214 specifications, evidencing a true dual-function ligninolytic–oleaginous phenotype	Al-Tohamy et al. (2021)

Abbreviations: MnP, manganese peroxidase; RB5, Reactive Black 5; AO7, Acid Orange 7; CDW, cell dry weight; SCO, Single-cell oil; NaCl, Sodium chloride; EN, 14214, European biodiesel quality standard; C16–C18, Fatty acids containing 16–18 carbon atoms.

### 5.4 Comparative benchmarking with conventional platforms

Termite-gut-derived yeasts have emerged as a strong alternative to traditional microbial platforms for bioremediation and biofuel production, primarily due to their unique enzymatic capabilities and resilience under challenging conditions. Compared to oleaginous yeasts such as *Y. lipolytica* and *Rhodotorula* spp., and white-rot fungi like *T. versicolor*, termite-associated yeasts offer distinct advantages, especially in the dual function of degrading lignin-derived aromatics and textile dyes while simultaneously producing lipids suitable for biodiesel generation (Ali et al., 2021a). This dual functionality not only positions these yeasts as promising candidates for integrated biorefinery systems, but also highlights their potential to couple waste remediation with renewable energy production. To enable a clear benchmark of performance across microbial platforms, Table 3 compares key metrics for ligninolytic/dye-degrading and oleaginous capabilities among termite-gut yeasts and conventional systems.

**TABLE 3:** Comparative benchmarking of microbial platforms for integrated aromatic pollutant degradation and biodiesel-oriented lipid production.

Organism	Enzymes	Dye range	Salinity tolerance	Lipid % DCW	Biodiesel profile	Scalability	References
*Meyerozyma caribbica* SSA1654	∼23–27 U/mL MnP	Sulfonated azo dyes (e.g., AO7) decolorisation 87%–98%	Tolerant to lignocellulose inhibitors (furfural, vanillin) but explicit NaCl data <50 g/L	∼47%	Cetane ∼52; favourable viscosity and cold-flow (modelled)	Lab-scale; termite-gut derived, promising but limited reactor data	Ali et al. (2022)
*Yarrowia lipolytica*	Laccase/oxidases present in some engineered strains; MnP rare	Conventional dyes when engineered; less data on refractory azo dyes	Generally low salinity tolerance in standard strains	∼30–50% achievable on sugars/glycerol	Good biodiesel profile in sugar-feed contexts	Commercially well known, but limited inherent ligninolytic/dye-degradation capacity	Sitepu et al. (2014)
*Rhodosporidium* (Rhodotorula) *toruloides*	Some oxidative enzyme reports; less MnP data	Phenolics, lignin fragments when adapted	Moderate salinity tolerance based on adaptation studies	∼40–70% DCW on optimized substrates	Good biodiesel profile (high monounsaturates)	Strong oleaginous platform; requires detoxified feedstocks	Lyu et al. (2021)
*Trametes versicolor*	High MnP/LiP/Lac reported	Wide aromatic substrate range including lignin, dyes	Often low salinity tolerance; strong requirement for optimal conditions	Lipid production not primary aim; thus % DCW low for biodiesel use	Biodiesel profile rarely reported for lipid from these fungi	Strong degradation but poor lipid integration; scale-up still challenging	Janusz et al. (2017)
Termite-gut yeast consortium (e.g., NYC-1)	Consortium multi-enzyme (MnP, Lac-like, azoreductase)	Mixed azo dyes + lignin-derived aromatics, rapid kinetics	Moderate salinity tolerance shown; full NaCl range not always specified	>40% on mixed substrates	Modelled biodiesel properties within EN 14214 limits	Promising integrated platform; yet scale-up and continuous operation not demonstrated	Ali et al. (2021a)

Abbreviations: Lac, Laccase; LiP, lignin peroxidase; MnP, manganese peroxidase; CDW, cell dry weight; AO7, Acid Orange 7.

One of the primary strengths of termite-gut yeasts lies in their resilience to inhibitory environmental conditions. These yeasts are notably more robust than conventional systems such as oleaginous yeasts, which often struggle with pre-treated, low-inhibitor substrates. For instance, *S. halophilus* SSA1575, a halotolerant termite-gut-derived yeast, has demonstrated high efficiency in decolorizing azo dyes even under high salinity and across a broad pH range (Al-Tohamy et al., 2020a). This is in stark contrast to many oleaginous yeasts, which generally require pre-treated, low-inhibitor feedstocks and are less suited for untreated industrial effluents, especially those containing lignin-derived phenolics or high levels of inorganic ions (Ali et al., 2021b). Further, when comparing enzyme portfolios, termite-gut yeasts exhibit an exceptional capacity for lignin degradation. For example, *M. caribbica* SSA1654, a MnP-producing oleaginous yeast, showed high extracellular MnP activity (∼23–27 U/mL) during decolorization of Acid Orange 7, a sulfonated azo dye, while simultaneously producing lipids suitable for biodiesel production (Ali et al., 2022). Conventional white-rot fungi like *T. versicolor* also express ligninolytic enzymes, but they typically require high moisture levels and controlled growth conditions, which limits their scalability and performance (Janusz et al., 2020). Additionally, termite-gut yeasts such as *S. halophilus* SSA1575 and *M. caribbica* SSA1654 have demonstrated significant tolerance to environmental stressors, such as high salinity, low pH, and toxic aromatic compounds, making them more effective in real effluent-like conditions. They outperform traditional microbial systems that typically struggle under similar conditions. This remarkable environmental resilience is one of the factors that set termite-derived yeasts apart and make them more suitable for large-scale bioremediation and biofuel production in industrial settings.

In terms of industrial application, while white-rot fungi have been extensively used in environmental remediation, their slow growth rates and high operational costs limit their scalability (Latif et al., 2023). In contrast, termite-gut yeasts offer faster growth rates and are more cost-effective in treating untreated industrial effluents, which contain complex mixtures of phenolic compounds, dyes, and salts (Ali et al., 2022; Al-Tohamy et al., 2020b). Furthermore, termite-derived yeasts can operate efficiently with untreated feedstocks and recalcitrant waste, positioning them as ideal candidates for integration into waste-to-energy biorefineries. Despite their potential, termite-derived yeasts still face challenges related to scale-up and industrial viability. Conventional oleaginous yeasts tend to perform better under pre-treated, low-inhibitor conditions, requiring complex pretreatment and nutrient supplementation to achieve optimal performance (Banner et al., 2021). However, the rapid growth, high tolerance to environmental stress, and efficient bioremediation capabilities of termite-derived yeasts suggest that these strains hold significant promise for large-scale industrial processes. The dual functionality of these yeasts—degrading pollutants while producing lipids for biofuel generation—offers a circular bioeconomy opportunity, making them highly attractive for sustainable industrial biorefinery applications.

Despite the promising attributes of termite-gut yeasts and their consortia, several key limitations constrain their translation toward industrial deployment. First, genomic and metabolic engineering studies remain scarce: while termite-gut systems are increasingly studied, few have complete genome sequences or genome editing frameworks established. For example, although the termite gut microbiome has been explored *via* metagenome-assembled genomes, the focus remains heavily on bacteria rather than oleaginous yeasts (Salgado et al., 2024). Secondly, bioreactor-scale demonstrations are lacking: the majority of research remains at the flask or lab-bench scale, and there is limited evidence of continuous flow systems using real industrial effluents and termite-derived yeasts. Third, cofactor regeneration constraints pose functional bottlenecks: high-redox oxidative enzymes (e.g., MnP, LiP) and azoreductases require cofactors such as H_2_O_2_ and NAD(P)H, and sustaining efficient cofactor recycling under complex effluent conditions is rarely addressed. Fourth, the field suffers from limited multi-omics integration: data sets combining genomics, transcriptomics, proteomics and metabolomics for termite-gut yeasts during aromatic pollutant stress and lipid accumulation remain virtually nonexistent, limiting mechanistic understanding of regulatory networks. Fifth, regulatory networks remain unknown: the signal transduction, stress response and metabolic re-routing that enable simultaneous aromatic degradation and lipid biosynthesis in termite-gut yeasts have not been elucidated. Finally, effluent variability and complexity pose real-world challenges: industrial wastewaters contain fluctuating pH, high ionic strength, heavy metals, mixed dye formulations and lignin fragments—yet many studies use simplified model substrates that fail to replicate this complexity (Xie et al., 2024).

To advance termite-gut yeast platforms toward industrial readiness, several strategic directions are recommended. Whole-genome sequencing, comparative genomics and establishment of genetic tools for termite-gut yeasts will facilitate the identification and enhancement of traits such as aromatic-polymer tolerance, stress workshops, and lipid flux rerouting. Bioreactor trials using real textile and lignin-rich effluents in continuous or semi-continuous configurations should prioritise scale-up, oxygen-transfer, cofactor regeneration, shear stress resilience and long-term stability. Integration of cofactor regeneration systems (e.g., engineered NADH-regeneration, H_2_O_2_-independent peroxidases) is critical to sustain high enzyme activity under industrial loads. Employing multi-omics (genome, transcriptome, proteome, metabolome) will enable mapping of regulatory networks linking pollutant transformation with lipid accumulation, thereby enabling rational strain design and process control. In parallel, TEA and LCA should be embedded early in development to assess circular-bioeconomy value, energy balance, carbon footprint and cost competitiveness. Finally, broadening substrate scope to real mixed waste streams (e.g., textile effluents + lignin hydrolysates + salts + heavy metals) will test system robustness and reveal bottlenecks, bringing termite-gut yeast platforms closer to full-scale reality.

## 6 Molecular aspects of aromatic waste bioremediation by yeasts

Recent advances in molecular biology and omics technologies have significantly expanded our understanding of how yeasts transform lignin-derived aromatics, synthetic azo dyes, and other industrial pollutants. High-throughput approaches—including whole-genome sequencing, transcriptomics, proteomics, and metagenomics—now enable the identification of key catabolic pathways, regulatory networks, and stress-response systems that underpin robust biodegradation performance (Jhariya et al., 2022). Applying these tools to pollutant-tolerant yeasts is essential for developing next-generation microbial platforms capable of operating in complex, inhibitor-rich industrial effluents (Danouche et al., 2021).

Despite extensive research on bacterial and fungal dye degradation, the molecular mechanisms governing yeast-based azo dye biotransformation remain comparatively underexplored. However, emerging evidence suggests that yeasts regulate a coordinated set of oxidative and reductive enzymes, membrane transporters, and stress-response systems during exposure to aromatic xenobiotics. Yeast surface display technology has become an important tool in this field, providing a powerful system for protein engineering and directed evolution. Because yeasts possess eukaryotic secretory pathways that support oxidative protein folding and glycosylation, they are highly suitable hosts for displaying complex enzymes, receptors, and multi-domain proteins on their cell surface (Tabañag et al., 2018; Thak et al., 2020). This enables rapid screening and engineering of mutants with enhanced stability, catalytic efficiency, and substrate specificity—traits that are crucial for degrading recalcitrant pollutants.

A wide range of functional proteins, including growth factors, antibody fragments, and membrane receptors, have been successfully displayed on yeast surfaces, demonstrating the versatility of this platform (Gai and Wittrup, 2007). The approach offers major technical advantages: (i) screening of large mutant libraries *via* fluorescence-activated cell sorting, (ii) minimizing host-expression bias through co-expression labeling, and (iii) direct evaluation of enzymatic properties without purification steps (Đurđić et al., 2020). These capabilities make yeast surface display particularly well suited for engineering oxidoreductases and peroxidases involved in aromatic pollutant degradation.

**FIGURE 3:**
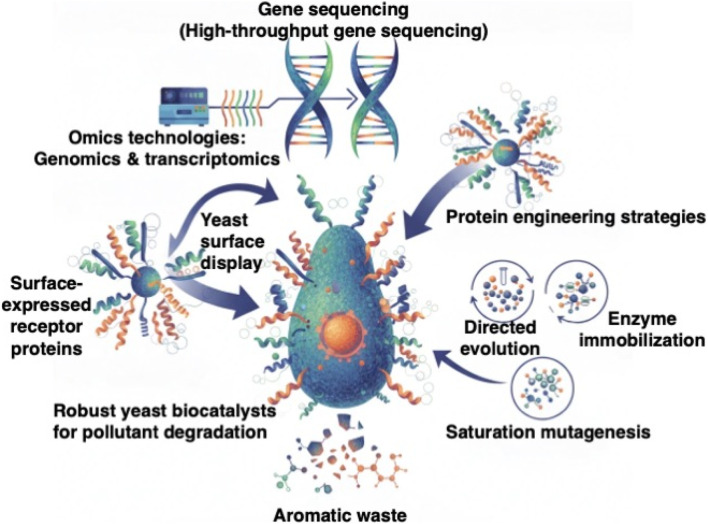
Molecular tools enhancing yeast-mediated aromatic waste bioremediation, illustrating how omics-guided enzyme discovery, yeast surface display, directed evolution, and protein-engineering strategies—including enzyme immobilization and saturation mutagenesis—are applied to engineer robust yeast biocatalysts capable of degrading lignin-derived aromatics and synthetic dyes. These engineered yeasts express optimized oxidative and reductive enzymes that accelerate aromatic-pollutant detoxification and channel the resulting intermediates into value-added metabolic pathways.

Molecular studies have also highlighted the importance of stress-responsive gene regulation in enhancing dye degradation. In a transcriptomic investigation, Wang et al. (2021) demonstrated that yeast strains capable of decolorizing azo dyes under saline conditions upregulated genes involved in glycerol biosynthesis, ion homeostasis, and remodeling of cell wall polysaccharides. These responses collectively strengthened halotolerance while improving enzymatic and non-enzymatic dye transformation. Similar studies have emphasized the role of protein engineering—via directed evolution, rational mutagenesis, and domain swapping—in enhancing the activity, stability, or cofactor affinity of dye-degrading enzymes (Jhariya et al., 2022; Aragaw et al., 2024b). These combined omics, surface-display, and protein-engineering approaches are summarized in Figure 3, which illustrates the molecular tools enabling the development of robust yeast biocatalysts for aromatic waste degradation.

Enzyme immobilization has traditionally been used to improve stability and reusability of oxidative enzymes. Immobilized peroxidases and laccases often retain catalytic activity across multiple cycles, but reduced conformational flexibility can impede electron transfer and lower turnover rates (Du et al., 2020; Abdelhamid et al., 2024). Yeast cell-surface display offers a practical alternative: anchoring oxidoreductases onto the external cell wall provides the functional advantages of immobilization while ensuring continuous metabolic support from the host cell. Yeasts are also Generally Recognized As Safe (GRAS), easily engineered, and capable of post-translational modifications—attributes that strengthen their suitability for whole-cell biocatalysis (Ye et al., 2021).

Recent protein-engineering efforts further highlight the potential of integrating recombinant enzymes into yeast-based remediation platforms. A recombinant peroxidase engineered through gene cloning achieved enhanced decolorization across several dye classes (Huy et al., 2021). Saturation mutagenesis and subsequent immobilization of an engineered versatile peroxidase on yeast surfaces allowed retention of high catalytic activity over repeated cycles, demonstrating its robustness in process-like conditions (Đurđić et al., 2020). Such results underline the feasibility of tailoring oxidative enzymes to withstand the physicochemical stresses found in textile and pulp-and-paper effluents.

Within the context of termite-gut-derived yeasts, these molecular tools are particularly relevant because several strains already combine desirable catalytic and physiological traits in a single chassis. MnP-producing oleaginous strains such as *M. caribbica* SSA1654, *M. guilliermondii* SSA1547, *Vanrija humicola* SSA1514 and *Debaryomyces hansenii* SSA1502 co-express ligninolytic peroxidases, azoreductases, glycosidases and lipases while tolerating lignocellulose-derived inhibitors and sustaining lipid accumulation. This native co-occurrence of stress tolerance and multi-enzyme activity reduces the engineering burden compared with transferring ligninolytic pathways into sensitive laboratory strains and identifies termite-derived yeasts as promising direct hosts for further genomic and regulatory optimization (Al-Tohamy et al., 2021).

Existing omics-enabled and protein engineering strategies can now be logically directed toward these termite-associated platforms. Prioritized targets include: (i) cloning and characterization of MnP and auxiliary oxidoreductase genes from strains such as SSA1654 to resolve structure–function features underlying their activity under inhibitor- and salt-rich conditions; (ii) promoter and regulatory engineering to decouple ligninolytic enzyme expression from growth limitations and increase volumetric activities on real effluents; and (iii) construction of defined synthetic consortia based on termite-derived yeasts with complementary MnP/laccase/azoreductase and lipid biosynthesis profiles, informed by NYC-1-type systems that already demonstrate stable, high-rate azo dye decolorization and biodiesel-compatible lipid profiles under process-relevant stresses. Integrating such termite-inspired genetic modules and regulatory circuits with established genome-editing tools (e.g., CRISPR–Cas systems, modular expression cassettes, cell-surface display) provides a rational route to next-generation microbial cell factories specifically tailored for lignin-based pollutant detoxification and lipid generation in integrated biorefineries.

## 7 Comparative evaluation of termite gut yeasts and conventional microbial platforms

Comparative evaluation of microbial platforms is essential to delineate the distinctive metabolic and enzymatic features that underpin their suitability for integrated bioremediation and biofuel production. This section synthesizes current evidence comparing termite-gut-derived oleaginous yeasts with conventional oleaginous yeasts, bacteria, and filamentous fungi, focusing on substrate tolerance, oxidative enzymology, lipid accumulation, pollutant-degradation performance, and process-integration potential. The key trends are summarized in Table 4, which highlights how termite-gut yeasts combine adaptive metabolism and robust oxidative enzymes to support dual functionality in pollutant detoxification and lipid biosynthesis.

**TABLE 4:** Performance of microbial platforms used for integrated bioremediation and biodiesel production.

Criterion	Conventional oleaginous yeasts	Bacterial and fungal biocatalysts	Termite gut yeasts	References
Substrate/inhibitor tolerance	Efficient on simple sugars, glycerol, some waste streams; growth and lipid synthesis often inhibited by furfural, HMF, and phenolics at modest concentrations	Many strains tolerate broader pollutant ranges but often prioritize degradation over lipid storage; lipid contents frequently below those of oleaginous yeasts	Isolates from termite gut tolerate azo dyes and lignocellulose-derived inhibitors while maintaining growth and lipid accumulation, reflecting adaptation to lignin-rich environments	El Kantar et al. (2021) Ali et al. (2021b); Sitepu et al. (2014)
Key oxidative/ligninolytic enzymes	LiP, MnP, laccase reported in some strains but typically with moderate stability (e.g., *Yarrowia lipolytica*, *Rhodosporidium toruloides*); often require optimized conditions and are not central to process design	Strong LiP/MnP/Lac activities in many ligninolytic fungi and bacteria; widely used for pollutant degradation but not primarily oleaginous	MnP- and peroxidase-producing termite-gut yeasts (e.g., *Meyerozyma caribbica* SSA1654) support azo dye and aromatic degradation linked to lipid biosynthesis, enabling dual functionality	Silva et al. (2023); Ali et al. (2021b); Janusz et al. (2017)
Lipid accumulation (typical % DCW)	Commonly about 20%–40%; engineered or optimized strains can exceed 50%–60% under ideal conditions and clean substrates	Frequently in the range of 10%–30% under practical or stress conditions	Approximately 40%–50% reported under dye/inhibitor stress for SSA1654 and related strains, demonstrating competitive lipid formation in contaminated matrices	Szczepańska et al. (2022), Ali et al. (2021b); Sitepu et al. (2014)
Pollutant degradation performance	Capable of some co-metabolic degradation, but aromatic detoxification is rarely the primary function; often requires prior detoxification steps	High degradation rates for dyes and phenolics in specialized ligninolytic fungi and bacteria; lipid accumulation generally low	Demonstrated decolorization and degradation of azo dyes and tolerance to lignin-derived inhibitors concurrent with lipid production, supporting integrated bioremediation–biodiesel schemes	Kumar et al. (2024); Ali et al. (2021b); Tan et al. (2019)
Process integration	Typically implemented in multi-step configurations: pretreatment/detoxification → lipid fermentation → downstream processing	Mainly applied in stand-alone bioremediation; coupling to lipid production is uncommon	Enables conceptual single-reactor processes where pollutant removal and lipid accumulation occur in the same culture, reducing unit operations	Lopes da Silva et al. (2023); Ali et al. (2021b); Costa et al. (2024)
Scalability and sustainability outlook	Strong industrial experience and genetic tools; TEA and LCA studies indicate promise but highlight costs of pretreatment and downstream steps	Established for wastewater and pollutant treatment; economic feasibility for combined fuel production less favorable due to lower lipid yields	Early-stage; lab-scale data support feasibility, but dedicated TEA/LCA, stability studies and pilot trials are still required to validate competitiveness	Sae-Ngae et al. (2020); Lopes da Silva et al. (2023); Ali et al. (2021b); Osman et al. (2024)

Abbreviations: Lac, Laccase; LiP, lignin peroxidase; MnP, manganese peroxidase; CDW, cell dry weight; HMF, 5-Hydroxymethylfurfural; TEA, techno-economic analysis; LCA, life-cycle assessment.

Conventional oleaginous yeasts such as *Y. lipolytica* and *Rhodosporidium toruloides* are well established as microbial oil producers and can reach high lipid contents under optimized conditions. Reviews report typical ranges of about 20%–40% DCW for many wild-type strains, with engineered or highly optimized strains reaching higher values, occasionally above 60% DCW under favorable substrates and process conditions (Ghogare et al., 2020). However, these systems are usually developed on relatively clean carbon sources (e.g., glucose, glycerol, agro-industrial side-streams) and are sensitive to lignocellulose-derived inhibitors such as furfural, 5-hydroxymethylfurfural, and phenolic compounds, which can severely impair growth and lipid formation (El Kantar et al., 2021). This sensitivity constrains their direct application in highly contaminated or aromatic-rich waste streams unless an additional detoxification step is implemented.

In contrast, termite-gut-derived yeasts have emerged as promising dual-function biocatalysts specifically adapted to lignocellulosic and aromatic-rich niches. *Meyerozyma caribbica* SSA1654, isolated from wood-feeding termite gut symbionts, has been shown to tolerate and transform azo dyes and lignocellulose-derived inhibitors while concurrently accumulating lipids at levels comparable to or exceeding several classical oleaginous yeasts (Ali et al., 2021b). Reports indicate that this strain maintains high lipid productivity in the presence of dye and inhibitor stress and exhibits MnP activity associated with oxidative degradation of aromatic compounds, directly linking pollutant removal with lipid biosynthesis in a single biological system (Ali et al., 2021a). This adaptive phenotype reflects the selective pressure of the termite gut environment and underpins the distinctive positioning of termite-associated yeasts for integrated bioremediation–biodiesel schemes.

In this context, the microbial biorefinery framework proposed by Al Azad et al. (2024) provides a valuable conceptual foundation for integrating regulatory, environmental, and economic considerations into the design of yeast-based remediation systems. Their work emphasizes that sustainable microbial biorefineries must balance waste valorization and energy recovery with biosafety and life-cycle assessment, ensuring that new biocatalysts—such as termite-gut yeasts—operate within environmentally responsible and legally compliant boundaries. Embedding termite-gut yeast applications within this holistic model could therefore accelerate their transition from laboratory innovation to industrial reality under a globally harmonized sustainability strategy.

A critical point of differentiation is process integration. Conventional microbial oil platforms typically rely on a modular flowsheet: physicochemical or biological detoxification of the feedstock, followed by cultivation of oleaginous microbes on the pretreated stream, and then lipid extraction and conversion. Comprehensive analyses of oleaginous yeast biorefineries emphasize that feedstock pretreatment, multi-step processing, and downstream separation substantially contribute to production costs and environmental footprints, and that TEA and LCA studies—while increasing—remain limited (Lopes da Silva et al., 2023). Termite-gut-derived yeasts offer a conceptual alternative by combining oxidative degradation and lipid formation in one organism, enabling a potential single-reactor configuration where aromatic pollutants act both as targets for detoxification and as contributors (directly or indirectly) to carbon flux toward lipids. Experimental demonstrations with *M. caribbica* SSA1654 and related termite-gut strains support this dual-function model at lab scale, suggesting a route to reduce unit operations, energy demand, and waste handling (Ali et al., 2021a).

However, a balanced assessment requires acknowledging the current limitations of termite-gut yeast systems. First, although termite-derived strains can achieve high lipid contents under stress (with values on the order of 40%–50% DCW reported for *M. caribbica* SSA1654 under dye or inhibitor exposure) (Ali et al., 2021b), these titers are not yet consistently superior to the best-performing engineered *Y. lipolytica* or other oleaginous yeasts cultivated on optimized substrates, which can surpass 60% DCW. Second, long-term genetic and metabolic stability of termite-gut isolates in continuous or industrially relevant conditions has not been thoroughly characterized. Third, while LCA and techno-economic frameworks have been developed for generic single-cell oil processes and oleaginous yeast biorefineries (Lopes da Silva et al., 2023), analogous assessments specifically tailored to termite-gut yeast-based systems are lacking. Finally, regulatory and biosafety considerations surrounding the deployment of gut-derived strains in open or semi-open treatment systems remain largely unexplored.

Taken together, the comparative evidence indicates that termite-gut yeasts should currently be viewed as complementary, rather than replacement, platforms to conventional oleaginous microbes. Their principal advantage lies in their capacity to endure and transform complex aromatic matrices while simultaneously generating lipids, thereby bridging the gap between pollutant-degradation-focused systems and high-yield lipid production platforms. In integrated lignin- and dye-rich waste valorization scenarios, this dual functionality can offer a strategic edge, particularly within circular bioeconomy frameworks that prioritize both environmental remediation and energy recovery. Realizing this potential will depend on targeted strain improvement, systematic kinetic and stability studies, and robust techno-economic and environmental assessments at pilot and demonstration scales to validate their competitiveness within the broader microbial biorefinery landscape.

## 8 Biomimetic and integrated biorefinery concept

**FIGURE 4:**
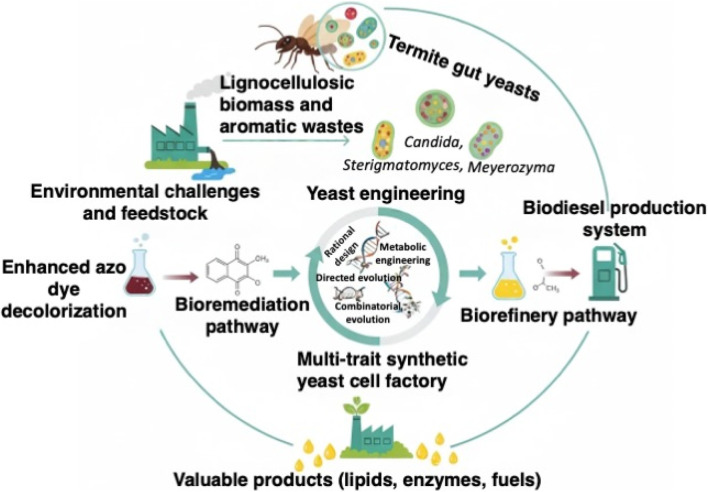
Integration of bioremediation and biorefinery technologies for the valorization of lignin-based aromatic waste, illustrating how termite-gut yeasts and associated ligninolytic taxa (e.g., *Candida*, *Sterigmatomyces*, *Meyerozyma*) can be engineered through omics-guided strategies to generate multi-trait synthetic yeast cell factories. These engineered strains couple aromatic-waste detoxification with lipid and metabolite biosynthesis, enabling enhanced azo-dye decolorization in the bioremediation pathway and biodiesel or lipid production in the biorefinery pathway, ultimately yielding valuable bioproducts.

The development of sustainable biorefinery systems capable of valorizing lignin-based aromatics and textile dyes requires biological platforms that efficiently couple bioremediation with lipid biosynthesis. Oleaginous yeasts have therefore attracted considerable attention as flexible microbial catalysts for converting waste-derived aromatic carbon into energy-rich lipids (Cho and Park, 2018; Bao et al., 2021; Robles-Iglesias et al., 2023). This biotechnological direction is driven by the urgent need to shift away from costly sugar-based fermentation substrates toward more abundant and refractory lignocellulose-derived streams, including both lignin fractions from agricultural residues and aromatic dyes from textile effluents (Ali et al., 2021b). Such integration of pollutant remediation with lipid production aligns seamlessly with global circular bioeconomy strategies that aim to simultaneously address waste mitigation and renewable energy generation. This integrated concept is illustrated in Figure 4, which outlines how termite-associated yeasts can be engineered into multi-trait biocatalysts that simultaneously degrade lignin-derived aromatics and channel metabolic intermediates toward biorefinery outputs.

Wood-feeding termites provide a compelling biomimetic model for designing such integrated systems. Their digestive tract represents one of nature’s most efficient lignocellulose-processing environments, combining mechanical disruption, selective lignin modification and microbial fermentation in a spatially organized, multi-step process. In contrast to industrial pretreatments that rely on harsh chemicals to achieve complete lignin depolymerization, termites selectively modify lignin in ways that enhance polysaccharide accessibility while minimizing chemical and energetic input (Sun et al., 2013; Kumar, 2018). These biological strategies highlight the value of adopting termite-inspired pretreatment principles—particularly the use of controlled oxidative modifications—within next-generation biorefineries. Enzymes and microbial partners derived from termite gut systems, including yeasts, bacteria and protozoa, thus offer unique opportunities for improving lignocellulose conversion and aromatic waste processing (Show et al., 2022).

Termite-associated yeasts represent an especially promising extension of this biomimetic framework. These yeasts naturally inhabit an environment rich in lignin derivatives, phenolics, salts and variable redox states, and therefore have evolved enzymatic and physiological features ideally suited for integrated aromatic remediation and lipid biosynthesis. Several studies have demonstrated that termite-derived yeasts can simultaneously transform azo dyes, degrade lignin-based aromatics and accumulate intracellular lipids that meet biodiesel quality requirements (Al-Tohamy et al., 2020a; Al-Tohamy et al., 2021; Ali et al., 2020; Ali et al., 2022). Their dual-function capabilities—stemming from stress-tolerant ligninolytic enzymes, reductases and robust oleaginous metabolism—offer a microbial route for converting complex effluents into renewable fuels (Figure 4).

Insights from termite digestion have also inspired new approaches for biomass pretreatment and valorization beyond the performance of individual strains. Recent biomimetic designs adopt principles derived from termite gut architecture, including staged oxidation, mild physicochemical conditions and enzyme synergism, to improve downstream hydrolysis and fermentation efficiency (Chen and Davaritouchaee, 2023). When these strategies are paired with robust termite-derived yeasts, biorefineries gain the ability not only to selectively depolymerize lignin but also to metabolize the resulting aromatics, achieving a continuous valorization of both carbohydrate and aromatic carbon streams. This dual-route valorization provides a solution to long-standing constraints in lignin utilization, where high pretreatment costs and inhibitor generation have hindered industrial implementation.

The pressing global need for sustainable waste management further underscores the importance of such integrated systems. With global waste generation expected to surpass 2.6 billion tons by 2025 (Sharma and Jain, 2020), valorizing lignin-rich industrial residues becomes a critical environmental priority. Traditional disposal methods, such as composting and incineration, generate significant greenhouse gases or secondary pollutants. In contrast, oleaginous yeasts offer a biological route for transforming inhibitory lignocellulose hydrolysates and dye-laden wastewaters into high-value lipids. Studies on *Rhodosporidium*, *Lipomyces* and *Cryptococcus* species have already demonstrated the potential of converting inhibitor-rich hydrolysates into single-cell oils while metabolizing compounds such as furfural, 5-HMF and acetic acid (Di Fidio et al., 2019; Siwina and Leesing, 2021). The naturally enhanced inhibitor tolerance and ligninolytic capacity of termite-derived yeasts further strengthen this vision, providing an evolutionary advantage in processing aromatic-rich substrates compared with conventional oleaginous strains. Within this broader technological context, termite-gut yeasts such as *S. halophilus*, *M. caribbica* and *M. guilliermondii* emerge as ideal candidates for next-generation biorefineries. Their performance in degrading azo dyes under saline or inhibitor-rich conditions, coupled with their ability to accumulate biodiesel-compatible lipids, demonstrates a direct operational pathway for integrating remediation and energy production within a single biological system (Al-Tohamy et al., 2020a; Al-Tohamy et al., 2021).

**FIGURE 5:**
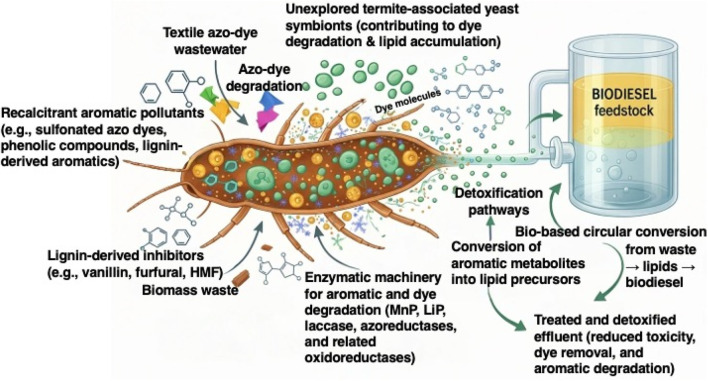
Conceptual model of wood-feeding termite gut symbionts involved in azo-dye degradation and lipid-precursor formation, illustrating how yeasts within the termite gut participate in the breakdown and detoxification of textile azo-dye wastewater, recalcitrant aromatic compounds, and lignin-derived inhibitors. Metabolic intermediates generated through these enzymatic degradation processes are subsequently funneled into pathways that produce lipid precursors suitable for biodiesel synthesis, highlighting the dual bioremediation and lipid-generating potential of termite-associated yeasts within circular bio-conversion systems.

Advances in synthetic biology and omics-driven metabolic engineering now provide the tools to refine and expand these termite-inspired platforms. Engineered strains that incorporate improved cofactor regeneration, enhanced MnP or laccase secretion, optimized lipid biosynthesis pathways or regulatory rewiring can increase both the rate and robustness of aromatic conversion and lipid accumulation (Elazzazy et al., 2025). The incorporation of termite-derived enzymes and regulatory elements into industrial hosts offers an additional route to strengthen performance under real-world conditions characterized by salinity, fluctuating pH and high aromatic load. Figure 5 illustrates the conceptual role of termite gut yeast symbionts in simultaneously degrading aromatic dye contaminants and converting resulting metabolites into lipid precursors suitable for biodiesel production. Ultimately, the convergence of biomimetic pretreatment strategies, stress-tolerant yeast biocatalysts and modern synthetic biology creates a coherent blueprint for future biorefineries capable of achieving the coupled goals of environmental detoxification and renewable biodiesel generation.

## 9 Engineering and economic outlook of termite-gut-yeast-based integrated biorefineries

The transition of termite-gut-derived yeasts from laboratory-scale demonstrations to industrially relevant integrated biorefineries demands a detailed understanding of engineering constraints and economic feasibility. Across microbial lipid platforms, TEA, LCA, and process systems engineering consistently identify substrate pretreatment, aeration and mixing energy, sterilization practices, and lipid recovery as dominant contributors to both operating costs and environmental impacts. Reviews of oleaginous-yeast biorefineries show that overall economic viability remains highly sensitive to feedstock price, energy consumption, and the degree of process integration (Silva et al., 2023). These observations form an essential reference point for evaluating termite-gut yeasts, whose dual ability to degrade aromatics and accumulate lipids may reduce reliance on conventional pretreatment and multi-stage processing.

TEA data from heterotrophic SCO processes illustrate the magnitude of economic challenges. Even under optimized fermentation conditions, projected minimum lipid selling prices typically range between 1.2 and 1.8 USD kg^−1^, while processes requiring extensive detoxification or sterilization can yield substantially higher costs (Karamerou et al., 2021). Similar trends have been reported for lignocellulosic biorefineries in which pretreatment severity, enzymatic hydrolysis, aeration demand, and intracellular lipid extraction dominate total expenditures. Economic performance improves markedly when low-cost residues, high-solids operations, and integrated resource-recovery strategies are implemented (Caporusso et al., 2022). These patterns underscore the need for TEA-tailored process designs for termite-gut-yeast systems, which remain at an early stage of development.

Termite-gut yeasts present two promising advantages: their tolerance to lignin-derived inhibitors and synthetic dyes may reduce detoxification requirements, and their ability to couple aromatic biodegradation with lipid accumulation could simplify flowsheets by consolidating multiple biological steps. Such biological integration has been proposed as a route to minimize unit operations and reduce aeration and heating requirements (Al-Tohamy et al., 2021). However, these advantages remain conceptual in the absence of TEA or LCA specific to termite-gut-yeast biorefineries, highlighting a major knowledge gap that must be addressed before claims of economic viability can be substantiated.

Engineering barriers must also be resolved to support scale-up. Stable lipid production in the presence of complex, inhibitor-rich effluents requires effective control of oxygen transfer, redox balance, nutrient supply, and mixing. Accumulation of partially oxidized aromatic intermediates can impair oxygen transfer, destabilize extracellular ligninolytic enzymes, or contribute to reactor fouling. Industrial systems also face challenges associated with reduced sterilization, which increases vulnerability to contamination and competition. TEA and LCA studies across microbial oil platforms highlight the need for integration of waste-heat recovery, optimized aeration strategies, *in situ* lipid extraction, and co-product valorization to offset energy and capital costs (Parsons et al., 2018).

Finally, the deployment of non-conventional yeasts—particularly those from insect microbiomes—introduces regulatory and biosafety considerations that influence engineering design. Pilot-scale demonstrations incorporating realistic effluents, supplemented by scenario-based TEA and LCA, will be essential to substantiate claims of scalability and to position termite-gut-derived yeasts credibly within next-generation microbial biorefineries.

## 10 Environmental sustainability and trade-offs in yeast-based integrated bioprocesses

Although yeast-based systems that integrate bioremediation with biodiesel production align conceptually with circular bioeconomy principles, their environmental sustainability depends on navigating several important trade-offs. Large-scale yeast cultivation requires sustained inputs of carbon, nutrients, and oxygen, and LCA and TEA studies repeatedly show that aeration, agitation, and lipid extraction dominate both energy use and environmental impact (Ochsenreither et al., 2016; Karamerou et al., 2021). Without careful energy integration, the electricity demands associated with maintaining high cell densities and intracellular lipid biosynthesis can erode or negate the greenhouse-gas benefits attributed to microbial biodiesel. Scenarios relying on low-cost aromatic-rich waste streams and employing optimized aeration and heat-recovery systems reduce the overall environmental burden, emphasizing that feedstock origin and process energy mix are critical determinants of sustainability (Mohiuddin et al., 2024).

Environmental risks also arise from the fate of pollutant-loaded yeast biomass. Biomass enriched with dyes, aromatic metabolites, or heavy metals can reintroduce contaminants into the environment if handled improperly. Studies on yeast-based wastewater treatment highlight the need for controlled biomass valorization—such as anaerobic digestion, stabilization, or recovery of lipids and proteins—to avoid secondary pollution while generating value-added products (Yang and Zheng, 2014). A further sustainability challenge concerns lipid extraction. Many SCO processes rely on organic solvents, whose production, use, and disposal contribute to environmental and health risks. Reviews of microbial lipid systems propose greener alternatives such as mechanical disruption, supercritical CO_2_ extraction, aqueous-phase separation, or *in situ* lipid recovery to minimize solvent-derived impacts (Ochsenreither et al., 2016; Karamerou et al., 2021).

Biological trade-offs within dual-function yeast platforms further complicate sustainability. Although oleaginous yeasts—including termite-gut-derived *M. caribbica* SSA1654—are capable of simultaneous dye degradation and lipid accumulation (Al-Tohamy et al., 2021), high lipid formation typically requires nitrogen limitation or stress conditions that slow growth and reduce extracellular enzyme production. TEA and kinetic analyses indicate that conditions maximizing lipid titers often differ from those favoring rapid and complete pollutant mineralization, implying that the two functions do not peak simultaneously (Ochsenreither et al., 2016; Karamerou et al., 2021). Because few current studies quantify these trade-offs explicitly, dual-function processes require integrated optimization rather than assuming that biodegradation and lipid accumulation are co-maximized.

To address these challenges, staged or compartmentalized process configurations are increasingly proposed. A first phase optimized for pollutant degradation can remove aromatic loads under conditions that maximize extracellular enzyme activities, followed by a second phase that redirects carbon toward lipid accumulation once toxicity is reduced. Concepts based on pollutant-loaded yeast biomass valorization and recent integrated biorefinery designs support this approach as a viable strategy for preserving treatment performance while enabling resource recovery (Yang and Zheng, 2014; Mohiuddin et al., 2024). Metabolic engineering and synthetic biology offer additional strategies to decouple degradation pathways from lipid biosynthesis, particularly in inherently stress-tolerant termite-gut-derived yeasts that can maintain performance under inhibitor-rich effluent conditions. Ultimately, robust LCA- and TEA-based assessments tailored specifically to dual-function yeast processes are required to quantify net greenhouse-gas savings, energy returns, solvent burdens, and biomass-handling impacts. Only through such evaluations can termite-gut-yeast-based biorefineries be positioned credibly as sustainable contributors to waste-to-resource systems.

## 11 Regulatory considerations and biosafety aspects of termite-gut-derived yeasts

The industrial application of termite-gut-derived yeasts—such as *M. caribbica* and *S. halophilus*—in bioremediation or integrated biorefineries must comply with biosafety and regulatory frameworks governing the intentional use of microorganisms. Although these yeasts are generally non-pathogenic and related to species used safely in food and environmental applications, their deployment in open or semi-open systems constitutes a potential environmental release requiring structured evaluation. OECD guidelines for environmental applications of microorganisms emphasize comprehensive characterization of any strain intended for release, including taxonomic identification, genomic stability, physiological traits, and ecological behavior, as well as its potential for survival, dispersal, and interactions with native biota (OECD, 2015). Recent analyses highlight that even naturally occurring, non-GM strains require environmental risk assessment (ERA) to evaluate potential unintended effects under realistic exposure scenarios (Lensch et al., 2023). For termite-gut yeasts, this necessitates genomic screening for virulence factors, mobile resistance genes, and secondary metabolite clusters, alongside assessment of growth at human-physiological temperatures.

Biosafety also depends on proper management of pollutant-loaded biomass. Data from yeast-based wastewater systems show that although selected yeasts effectively remove nutrients, metals, and dyes, spent biomass must be processed through controlled stabilization or valorization routes to avoid secondary contamination (Nicula et al., 2023). This requirement extends to systems degrading lignin-derived aromatics, where residual intermediates may pose environmental concerns. Reviews of yeast-mediated pollutant removal further emphasize that controlled recovery, immobilization, or destruction of biomass is essential to prevent desorption or leaching of contaminants during disposal (Shao et al., 2025).

Process configuration is another major regulatory consideration. Closed or semi-closed reactors, immobilization matrices, or encapsulation systems are widely recommended as best practice for limiting environmental dissemination, aligning with guidelines for intentional use of microorganisms in natural or engineered environments (OECD, 2015; Lensch et al., 2023). Immobilized systems are particularly advantageous for termite-gut-derived yeasts because they support reuse, monitoring, and controlled disposal. Where applications inherently involve partial environmental exposure—such as *in situ* effluent treatment—regulators expect data on persistence, competitiveness, and reversibility of strain establishment. If termite-gut-derived yeasts are engineered to enhance ligninolytic activity, stress tolerance, or lipid accumulation, they fall under genetically modified microorganism frameworks that require detailed molecular characterization, evaluation of horizontal gene transfer potential, and modeling of plausible exposure scenarios (Rubinstein et al., 2025). Contemporary perspectives emphasize genetic and physical containment tools—auxotrophic safeguards, kill-switches, dependency circuits, or strict confinement—as means to reduce risk and facilitate regulatory acceptance (Lea-Smith et al., 2025). Emerging microbial-biorefinery frameworks similarly emphasize standardized closed operation, biomass valorization, and integrated product recovery as strategies to strengthen environmental safety and regulatory compliance (Al Azad et al., 2024).

## 12 Conclusion

Termite-gut-derived yeasts represent a uniquely promising yet underexplored microbial resource for next-generation circular biorefineries that couple bioremediation with biodiesel production through integrated ligninolytic and oleaginous pathways. Their natural tolerance to lignin-derived aromatics, textile dyes, salinity, and other industrial stressors, combined with their capacity to express diverse oxidative and reductive enzymes while simultaneously accumulating high-value lipids, positions them as compelling dual-function biocatalysts capable of addressing persistent challenges in wastewater treatment and renewable fuel generation. Exemplary strains such as *M. caribbica* SSA1654 and *S. halophilus* SSA1575 consistently outperform many conventional oleaginous yeasts and filamentous fungi under inhibitor-rich conditions, offering operational advantages that could reduce pretreatment requirements, streamline process flowsheets, and expand the spectrum of industrial effluents usable as both remediation substrates and carbon sources. Despite this potential, the pathway to industrial deployment remains incomplete: the absence of high-quality genome assemblies, regulatory network maps, and multi-omics datasets limits rational strain engineering, while the lack of targeted metabolic optimization constrains improvements in ligninolytic expression, redox management, stress resilience, and lipid biosynthesis—traits central to resolving the trade-offs inherent in dual-function operation. At the process level, most studies remain confined to laboratory batch cultures, and the kinetic performance, enzyme stability, oxygen-transfer requirements, and long-term robustness of termite-gut yeasts in real effluents await validation through continuous or pilot-scale bioreactor trials. Their environmental and economic impacts likewise remain speculative in the absence of TEA and LCA tailored specifically to termite-yeast integrated biorefineries. Moreover, regulatory and biosafety considerations—including biomass-handling strategies, pollutant-loaded residue management, and the need for genetic or physical containment, particularly if engineered strains are used—must be addressed before industrial implementation. Moving forward, meaningful progress will require coordinated advances that integrate genomics, metabolic engineering, and synthetic biology with process intensification, reactor design, and systems-level sustainability assessment. The development of staged or compartmentalized cultivation schemes, improved cofactor-regeneration strategies, and designer microbial consortia may further enhance stability and productivity. As these scientific, engineering, and regulatory gaps are progressively closed, termite-gut yeasts are poised to advance from ecological curiosities to key catalysts within circular bioeconomy infrastructures, enabling the sustainable conversion of aromatic wastes into renewable fuels and value-added bioproducts while delivering tangible environmental and societal benefits.

## References

[B1] Abdelhamid M. A. Khalifa H. O. Yoon H. J. Ki M. R. Pack S. P. (2024). Microbial immobilized enzyme biocatalysts for multipollutant mitigation: harnessing nature’s toolkit for environmental sustainability. Int. J. Mol. Sci. 25, 8616. 10.3390/ijms25168616 39201301 PMC11355015

[B2] Abdi Dezfouli R. Esmaeilidezfouli E. (2024). Optimizing laccase selection for enhanced outcomes: a comprehensive review. 3 Biotech. 14, 165. 10.1007/s13205-024-04157-x 38817737 PMC11133268

[B3] Abdullah T. İlyasoğlu G. Memić A. (2023). Designing lignin-based biomaterials as carriers of bioactive molecules. Pharmaceutics 15, 1114. 10.3390/pharmaceutics15041114 37111600 PMC10143462

[B4] Affat S. S. (2021). Classifications, advantages, disadvantages, and toxicity effects of natural and synthetic dyes: a review. Univ. Thi-Qar J. Sci. 8, 130–135. Available online at: http://www.jsci.utq.edu.iq/index.php/main/article/view/790.

[B5] Akter T. Protity A. T. Shaha M. Al Mamun M. Hashem A. (2023). “The impact of textile dyes on the environment,” in Nanohybrid materials for treatment of textile dyes (Singapore: Springer Nature Singapore), 401–431.

[B6] Al Azad S. Madadi M. Song G. Sun C. Sun F. (2024). New trends in microbial lipid-based biorefinery for fermentative bioenergy production from lignocellulosic biomass. Biofuel Res. J. 11, 2040–2064. 10.18331/BRJ2024.11.1.5

[B7] Al-Gethami W. Qamar M. A. Shariq M. Alaghaz A. N. M. Farhan A. Areshi A. A. (2024). Emerging environmentally friendly bio-based nanocomposites for the efficient removal of dyes and micropollutants from wastewater by adsorption: a comprehensive review. RSC Adv. 14, 2804–2834. 10.1039/D3RA07411K 38234871 PMC10792434

[B8] Al-Tohamy R. Kenawy E. R. Sun J. Ali S. S. (2020a). Performance of a newly isolated salt-tolerant yeast strain *Sterigmatomyces halophilus* SSA-1575 for azo dye decolorization and detoxification. Front. Microbiol. 11, 1163. 10.3389/fmicb.2020.01163 32595618 PMC7300265

[B9] Al-Tohamy R. Sun J. Fareed M. F. Kenawy E. R. Ali S. S. (2020b). Ecofriendly biodegradation of reactive black 5 by newly isolated *Sterigmatomyces halophilus* SSA-1575, valued for textile azo dye wastewater processing and detoxification. Sci. Rep. 10, 12370. 10.1038/s41598-020-69278-0 32704008 PMC7378048

[B10] Al-Tohamy R. Sun J. Khalil M. A. Kornaros M. Ali S. S. (2021). Wood-feeding termite gut symbionts as an obscure yet promising source of novel manganese peroxidase-producing oleaginous yeasts intended for azo dye decolorization and biodiesel production. Biotechnol. Biofuels 14, 27. 10.1186/s13068-021-01971-y 34863263 PMC8645103

[B11] Al-Tohamy R. Ali S. S. Li F. Okasha K. M. Mahmoud Y. A. G. Elsamahy T. (2022). A critical review on the treatment of dye-containing wastewater: ecotoxicological and health concerns of textile dyes and possible remediation approaches for environmental safety. Ecotoxicol. Environ. Saf. 241, 113756. 10.1016/j.ecoenv.2021.113160 35026583

[B12] Al-Tohamy R. Ali S. S. Zhang M. Sameh M. Mahmoud Y. A. G. Waleed N. (2023a). Can wood-feeding termites solve the environmental bottleneck caused by plastics? A critical state-of-the-art review. J. Environ. Manag. 326, 116606. 10.1016/j.jenvman.2022.116606 36403319

[B13] Al-Tohamy R. Ali S. S. Xie R. Schagerl M. Khalil M. A. Sun J. (2023b). Decolorization of reactive azo dye using novel halotolerant yeast consortium HYC and proposed degradation pathway. Ecotoxicol. Environ. Saf. 263, 115258. 10.1016/j.ecoenv.2023.115258 37478569

[B14] Ali S. S. Al-Tohamy R. Sun J. Wu J. Huang M. (2018). The role of gut symbionts from termites: a unique hidden player from yeasts. Acta Microbiol. Sin. 58 (6), 1004–1015. 10.13343/j.cnki.wsxb.20170610

[B15] Ali S. S. Al-Tohamy R. Xie R. El-Sheekh M. M. Sun J. (2020). Construction of a new lipase- and xylanase-producing oleaginous yeast consortium capable of reactive azo dye degradation and detoxification. Bioresour. Technol. 313, 123631. 10.1016/j.biortech.2020.123631 32540694

[B16] Ali S. S. Al-Tohamy R. Koutra E. Kornaros M. Khalil M. Elsamahy T. (2021a). Coupling azo dye degradation and biodiesel production by manganese-dependent peroxidase producing oleaginous yeasts isolated from wood-feeding termite gut symbionts. Biotechnol. Biofuels 14, 61. 10.1186/s13068-021-01905-9 33685508 PMC7938474

[B17] Ali S. S. Al-Tohamy R. Koutra E. El-Naggar A. H. Kornaros M. Sun J. (2021b). Valorizing lignin-like dyes and textile dyeing wastewater by a newly constructed lipid-producing and lignin-modifying oleaginous yeast consortium valued for biodiesel and bioremediation. J. Hazard. Mater. 403, 123575. 10.1016/j.jhazmat.2020.123575 32791477

[B18] Ali S. S. Al-Tohamy R. Sun J. (2022). Performance of *Meyerozyma caribbica* as a novel manganese peroxidase-producing yeast inhabiting wood-feeding termite gut symbionts for azo dye decolorization and detoxification. Sci. Total Environ. 806, 150665. 10.1016/j.scitotenv.2021.150665 34597540

[B19] Ali S. S. Al-Tohamy R. Sun J. (2025). Disruptive technology for integrating bioremediation and biodiesel production from persistent toxic aromatic wastes using termite gut yeasts. Environ. Chem. Ecotoxicol. 7, 462–493. 10.1016/j.enceco.2025.01.004

[B20] Ali S. S. Amer O. A. Al-Zahrani M. (2026). “Lignin-based carbon fiber,” in Lignin-driven advanced materials (Amsterdam, Netherlands:Elsevier), 149–177. 10.1016/B978-0-323-85872-3.00010-2

[B21] Aragaw T. A. (2024). Potential and prospects of reductases in azo dye degradation: a review. Microbe 4, 100162. 10.1016/j.microbe.2023.100162

[B22] Aragaw T. A. Bogale F. M. Tesfaye E. L. (2024a). Ligninolytic oxidative enzymes and their role in textile dye biodegradation: a comprehensive review. Water Pract. Technol., 19, 3598–3630. 10.2166/wpt.2024.229

[B23] Aragaw T. A. Bogale F. M. Tesfaye E. L. (2024b). Oxidative ligninolytic enzymes and their role in textile dye biodegradation: a comprehensive review. Water Pract. Technol. 19, 3598–3630. 10.2166/wpt.2024.207

[B24] Atiwesh G. Parrish C. C. Banoub J. Le T. A. T. (2022). Lignin degradation by microorganisms: a review. Biotechnol. Prog. 38, e3226. 10.1002/btpr.3226 34854261

[B25] Ayele A. Godeto Y. G. (2021). Bioremediation of chromium by microorganisms and its mechanisms related to functional groups. J. Chem. 2021, 6678230. 10.1155/2021/6678230

[B26] Baker P. Tiroumalechetty A. Mohan R. (2019). “Fungal enzymes for bioremediation of xenobiotic compounds,” in Recent advancement in white biotechnology through fungi: volume 3 – perspective for sustainable environments (Cham, Switzerland: Springer International Publishing), 463–489. 10.1007/978-3-030-25506-0_19

[B27] Bala S. Garg D. Thirumalesh B. V. Sharma M. Sridhar K. Inbaraj B. S. (2022). Recent strategies for bioremediation of emerging pollutants: a review for a green and sustainable environment. Toxics 10, 484. 10.3390/toxics10080484 36006163 PMC9413587

[B28] Bandhu S. Srivastava A. Ghosh D. Chaudhuri T. K. (2020). Yeast single-cell oils from bioresources: current developments in production and applications. Curr. Sustain. Renew. Energy Rep. 7, 109–120. 10.1007/s40518-020-00161-3

[B29] Banner A. Toogood H. S. Scrutton N. S. (2021). Consolidated bioprocessing: synthetic biology routes to fuels and fine chemicals. Microorganisms 9, 1079. 10.3390/microorganisms9051079 34069865 PMC8157379

[B30] Bao W. Li Z. Wang X. Gao R. Zhou X. Cheng S. (2021). Approaches to improve lipid synthesis of the oleaginous yeast *Yarrowia lipolytica*: a review. Renew. Sustain. Energy Rev. 149, 111386. 10.1016/j.rser.2021.111386

[B31] Barber-Zucker S. Mindel V. Garcia-Ruiz E. Weinstein J. J. Alcalde M. Fleishman S. J. (2022). Stable and functionally diverse versatile peroxidases designed directly from sequences. J. Am. Chem. Soc. 144, 3564–3571. 10.1021/jacs.1c10626 35179866 PMC8895400

[B32] Benkhaya S. M’rabet S. El Harfi A. (2020). Classifications, properties, recent synthesis and applications of azo dyes. Heliyon 6, e03271. 10.1016/j.heliyon.2020.e03271 32042981 PMC7002841

[B33] Bhuvaneswari M. Subashini R. Crossia J. W. F. Vijayalakshmi S. (2020). “Mycoremediation of industrial dyes by laccases,” in New and future developments in microbial biotechnology and bioengineering (Amsterdam, Netherlands: Elsevier), 235–243. 10.1016/B978-0-12-821010-4.00013-2

[B34] Biko O. D. Viljoen-Bloom M. van Zyl W. H. (2020). Microbial lignin peroxidases: applications, production challenges, and future perspectives. Enzyme Microb. Technol. 141, 109669. 10.1016/j.enzmictec.2020.109669 33051019

[B35] Blasi A. Verardi A. Lopresto C. G. Siciliano S. Sangiorgio P. (2023). Lignocellulosic agricultural waste valorization to obtain valuable products: an overview. Recycling 8, 61. 10.3390/recycling8040061

[B36] Broos W. Wittner N. Geerts J. Dries J. Vlaeminck S. E. Gunde-Cimerman N. (2022). Evaluation of lignocellulosic wastewater valorization with the oleaginous yeasts *R. kratochvilovae* EXF7516 and *C. oleaginosum* ATCC 20509. Fermentation 8, 204. 10.3390/fermentation8050204

[B37] Brune A. (2014). Symbiotic digestion of lignocellulose in termite guts. Nat. Rev. Microbiol. 12, 168–180. 10.1038/nrmicro3182 24487819

[B38] Cagide C. Castro-Sowinski S. (2020). Technological and biochemical features of lignin-degrading enzymes: a brief review. Environ. Sustain. 3, 371–389. 10.1007/s42398-020-00128-1

[B39] Calabon M. S. Hyde K. D. Jones E. B. G. Bao D. F. Bhunjun C. S. Phukhamsakda C. (2023). Freshwater fungal biology. Mycosphere 14, 195–413. 10.5943/mycosphere/14/1/11

[B40] Caporusso A. Giuliano A. Liuzzi F. De Bari I. (2022). Techno-economic analysis of a lignocellulosic biorefinery producing microbial oils by oleaginous yeasts. Chem. Eng. Trans. 92, 637–642. 10.3303/CET2292107

[B41] Chakraborty A. Šobotník J. Votýpková K. Hradecký J. Stiblik P. Synek J. (2023). Impact of wood age on termite microbial assemblages. Appl. Environ. Microbiol. 89, e00361-23. 10.1128/aem.00361-23 37067424 PMC10231148

[B42] Chen S. Davaritouchaee M. (2023). Nature-inspired pretreatment of lignocellulose: perspective and development. Bioresour. Technol. 369, 128456. 10.1016/j.biortech.2022.128456 36503090

[B43] Chequer F. D. De Oliveira G. R. Ferraz E. A. Cardoso J. C. Zanoni M. B. De Oliveira D. P. (2013). Textile dyes: dyeing process and environmental impact. Eco-friendly Text. Dye. Finish. 6 (6), 151–176. 10.5772/53659

[B44] Cho H. U. Park J. M. (2018). Biodiesel production by various oleaginous microorganisms from organic wastes. Bioresour. Technol. 256, 502–508. 10.1016/j.biortech.2018.01.153 29478783

[B45] Costa G. D. S. Martinez-Burgos W. J. dos Reis G. A. Puche Y. P. Vega F. R. Rodrigues C. (2024). Advances in biomass and microbial lipids production: trends and prospects. Processes 12, 2903. 10.3390/pr12122903

[B46] Danouche M. El Arroussi H. El Ghachtouli N. D. (2021). Mycoremediation of synthetic dyes by yeast cells: a sustainable biodegradation approach. Environ. Sustain 4, 5–22. 10.1007/s42398-020-00149-4

[B47] de Mello A. C. C. da Silva F. P. Gripa E. Salgado A. M. da Fonseca F. V. (2023). Phenol removal from wastewater using tyrosinase enzyme immobilized in granular activated carbon and activated chitosan beads. Water 15, 3778. 10.3390/w15213778

[B48] Di Fidio N. Liuzzi F. Mastrolitti S. Albergo R. De Bari I. (2019). Single cell oil production from undetoxified *Arundo donax* L. hydrolysate by *Cutaneotrichosporon curvatus* . J. Microbiol. Biotechnol. 29 (2), 256–267. 10.4014/jmb.1812.12004 30866181

[B49] Dobrowolski A. Drzymała K. Rzechonek D. A. Mituła P. Mirończuk A. M. (2019). Lipid production from waste materials in seawater-based medium by the yeast *Yarrowia lipolytica* . Front. Microbiol. 10, 547. 10.3389/fmicb.2019.00547 30936863 PMC6431633

[B50] Du H. Shi S. Liu W. Teng H. Piao M. (2020). Processing and modification of hydrogel and its application in emerging contaminant adsorption and catalyst immobilization: a review. Environ. Sci. Pollut. Res. 27, 12967–12994. 10.1007/s11356-020-07908-8 32124301

[B51] Dumond L. Lam L. P. Y. van Erven G. Kabel M. Mounet F. Grima-Pettenati J. (2021). Termite gut microbiota contribution to wheat straw delignification in anaerobic bioreactors. ACS Sustain. Chem. Eng. 9, 2191–2202. 10.1021/acssuschemeng.0c07309

[B52] Đurđić K. I. Ostafe R. Đelmaš A. Đ. Popović N. Schillberg S. Fischer R. (2020). Saturation mutagenesis to improve the degradation of azo dyes by versatile peroxidase and application in the form of VP-coated yeast cell walls. Enzyme Microb. Technol. 136, 109509. 10.1016/j.enzmictec.2020.109509 32331716

[B53] El Kantar S. Khelfa A. Vorobiev E. Koubaa M. (2021). Strategies for increasing lipid accumulation and recovery from *Yarrowia lipolytica*: a review. OCL 28, 51. 10.1051/ocl/2021040

[B54] Elazzazy A. M. Baeshen M. N. Alasmi K. M. Alqurashi S. I. Desouky S. E. Khattab S. M. (2025). Where biology meets engineering: scaling up microbial nutraceuticals to bridge nutrition, therapeutics, and global impact. Microorganisms 13 (3), 566. 10.3390/microorganisms13030566 40142459 PMC11945976

[B55] Emanuele L. D’Auria M. (2024). The use of heterocyclic azo dyes on different textile materials: a review. Organics 5, 277–289. 10.3390/org5030020

[B56] Gai S. A. Wittrup K. D. (2007). Yeast surface display for protein engineering and characterization. Curr. Opin. Struct. Biol. 17, 467–473. 10.1016/j.sbi.2007.07.001 17870469 PMC4038029

[B57] Ghogare R. Chen S. Xiong X. (2020). Metabolic engineering of the oleaginous yeast *Yarrowia lipolytica* for overproduction of fatty acids. Front. Microbiol. 11, 1717. 10.3389/fmicb.2020.01717 32849364 PMC7418586

[B58] Gouda S. A. Taha A. (2023). Biosorption of heavy metals as a new alternative method for wastewater treatment: a review. Egypt. J. Aquat. Biol. Fish. 27, 135–153. 10.21608/ejabf.2023.287177

[B59] Goux X. Liu T. Westerholm M. Calusinska M. (2023). “Microbial degradation of lignocellulose in natural and engineered systems—from the smallest to the biggest bioreactor,” in Microbial fermentations in nature and as designed processes, Editor Hurst C. J. (Hoboken, NJ: John Wiley and Sons). 167–205.

[B60] Huang H. Xu C. Zhu X. Li B. Huang C. (2023). Lignin-enhanced wet strength of cellulose-based materials: a sustainable approach. Green Chem. 25, 4995–5009. 10.1039/D3GC00705E

[B61] Huy N. D. Le N. T. M. Chew K. W. Park S. M. Show P. L. (2021). Characterization of a recombinant laccase from *Fusarium oxysporum* HUIB02 for biochemical application on dye removal. Biochem. Eng. J. 168, 107958. 10.1016/j.bej.2020.107958

[B62] Ibitoye F. O. Imarhiagbe E. E. Ekhaise F. O. (2022). A review on the ecological impacts of azo dye and survey of bioremediation potential strains. NIPES J. Sci. Technol. Res. 4, 1–12. 10.37933/nipes/4.3.2022.1

[B63] Ivanka S. Albert K. Veselin S. (2010). Properties of crude laccase from *Trametes versicolor* produced by solid-substrate fermentation. Adv. Biosci. Biotechnol. 1, 208–215. 10.4236/abb.2010.13029

[B64] Janusz G. Pawlik A. Sulej J. Świderska-Burek U. Jarosz-Wilkołazka A. Paszczyński A. (2017). Lignin degradation: microorganisms, enzymes involved, genome analysis, and evolution. FEMS Microbiol. Rev. 41, 941–962. 10.1093/femsre/fux049 29088355 PMC5812493

[B65] Janusz G. Pawlik A. Świderska-Burek U. Polak J. Sulej J. Jarosz-Wilkołazka A. (2020). Laccase properties, physiological functions, and evolution. Int. J. Mol. Sci. 21, 966. 10.3390/ijms21030966 32024019 PMC7036934

[B66] Jhariya U. Srivastava S. Das S. Bombaywala S. Mahalle S. Dafale N. A. (2022). “Understanding the role of genetic and protein networking involved in microbial bioremediation,” in Bioremediation of environmental pollutants: emerging trends and strategies (Amsterdam: Elsevier), 187–219. 10.1016/B978-0-323-89910-5.00011-5

[B67] Jiao H. Xiong M. Al-Tohamy R. Schagerl M. Kornaros M. Ali S. S. (2026). Rethinking plastics through microbial biodegradation and circular economy innovation: a review. Environ. Chem. Ecotoxicol. 8, 1995–2224. 10.1016/j.enceco.2025.11.009

[B68] Kanwal S. Sana H. Khan M. K. Mujahid R. Zeb H. (2024). “Biomass feedstock: a sustainable and renewable source of energy production,” in Nanomaterials in biomass conversion (Oxford, United Kingdom: Woodhead Publishing), 1–34. 10.1016/B978-0-323-90234-8.00001-0

[B69] Karamerou E. E. Parsons S. McManus M. C. Chuck C. J. (2021). Using techno-economic modelling to determine the minimum cost possible for a microbial palm oil substitute. Biotechnol. Biofuels 14, 57. 10.1186/s13068-021-01966-9 33663577 PMC7934523

[B70] Kaur A. Saini J. K. (2024). “Lignin-degrading enzymes: production and applications,” in Biomass hydrolyzing enzymes (Boca Raton, FL: CRC Press), 72–85.

[B71] Khaliq N. (2023). “Microbial enzymes as a robust process to mitigate pollutants of environmental concern,” in Microbial biomolecules (San Diego, CA: Academic Press), 241–267. 10.1016/B978-0-323-85770-9.00006-1

[B72] Kivevele T. T. Huan Z. (2013). Effects of antioxidants on the cetane number, viscosity, oxidation stability, and thermal properties of biodiesel produced from nonedible oils. Energy Technol. 1, 537–543. 10.1002/ente.201300054

[B73] Kolawole A. S. Iyiola A. O. (2023). “Environmental pollution: threats, impact on biodiversity, and protection strategies,” in Sustainable utilization and conservation of Africa’s biological resources and environment (Singapore: Springer), 377–409. 10.1007/978-981-99-1811-1_18

[B74] Kumar N. G. (2018). “Termites,” in Pests and their management (Singapore: Springer), 909–971. 10.1007/978-981-10-4329-8_29

[B75] Kumar A. Chandra R. (2020). Ligninolytic enzymes and their mechanisms for degradation of lignocellulosic waste in the environment. Heliyon 6, e03170. 10.1016/j.heliyon.2020.e03170 32095645 PMC7033530

[B76] Kumar V. Pallavi P. Sen S. K. Raut S. (2024). Harnessing the potential of white-rot fungi and ligninolytic enzymes for efficient textile dye degradation: a comprehensive review. Water Environ. Res. 96, e10959. 10.1002/wer.10959 38204323

[B77] Latif W. Ciniglia C. Iovinella M. Shafiq M. Papa S. (2023). Role of white rot fungi in industrial wastewater treatment: a review. Appl. Sci. 13, 8318. 10.3390/app13148318

[B78] Lea-Smith J. D. Hassard F. Coulon F. Partridge N. Horsfall L. Parker K. D. (2025). Engineering biology applications for environmental solutions: potential and challenges. Nat. Commun. 16, 3538. 10.1038/s41467-025-15338-2 40229265 PMC11997111

[B79] Ledakowicz S. Paździor K. (2021). Recent achievements in dyes removal focused on advanced oxidation processes integrated with biological methods. Molecules 26, 870. 10.3390/molecules26040870 33562176 PMC7914684

[B80] Lensch A. Lindfors H. A. Duwenig E. Fleischmann T. Hjort C. Kärenlampi S. O. (2023). Safety aspects of microorganisms deliberately released into the environment. EFB Bioecon. J. 4, 100061. 10.1016/j.bioeco.2023.100061

[B81] Lopes da Silva T. Fontes A. Reis A. Siva C. Gírio F. (2023). Oleaginous yeast biorefinery: feedstocks, processes, techniques, and bioproducts. Fermentation 9, 1013. 10.3390/fermentation9121013

[B82] Lyu L. Chu Y. Zhang S. Zhang Y. Huang Q. Wang S. (2021). Engineering the oleaginous yeast *Rhodosporidium toruloides* for improved resistance against inhibitors in biomass hydrolysates. Front. Bioeng. Biotechnol. 9, 768934. 10.3389/fbioe.2021.768934 34869282 PMC8634367

[B83] Lyu Q. Dar R. A. Baganz F. Smoliński A. Rasmey A. H. M. Liu R. (2025). Effects of lignocellulosic biomass-derived hydrolysate inhibitors on cell growth and lipid production during microbial fermentation of oleaginous microorganisms—A review. Fermentation 11, 121. 10.3390/fermentation11030121

[B84] Malassigné S. Minard G. Vallon L. Martin E. Valiente-Moro C. Luis P. (2021). Diversity and functions of yeast communities associated with insects. Microorganisms 9, 1552. 10.3390/microorganisms9081552 34442634 PMC8399037

[B85] Mehra S. Singh M. Chadha P. (2021). Adverse impact of textile dyes on the aquatic environment as well as on human beings. Toxicol. Int. 28, 165–176. 10.4103/toxint.toxint_25_21

[B86] Mohiuddin O. Harvey A. P. Ledesma M. T. O. Velasquez-Orta S. (2024). Bioremediation of waste by yeast strains. Electron. J. Biotechnol. 69, 30–42. 10.1016/j.ejbt.2024.01.003

[B87] Nath S. Naha A. Saikia K. Choudhury C. P. Venkatramanan V. (2025). Degradation of organic pollutants using lignin-derived carbon materials as a sustainable approach to environmental remediation. Biotechnol. Sustain. Mater. 2, 11. 10.1186/s44316-025-00036-z

[B88] Nicula N. O. Lungulescu E. M. Rîmbu G. A. Marinescu V. Corbu V. M. Csutak O. (2023). Bioremediation of wastewater using yeast strains: an assessment of contaminant removal efficiency. Int. J. Environ. Res. Public Health 20, 4795. 10.3390/ijerph20064795 36981703 PMC10048942

[B89] Ochsenreither K. Glück C. Stressler T. Fischer L. Syldatk C. (2016). Production strategies and applications of microbial single cell oils. Front. Microbiol. 7, 1539. 10.3389/fmicb.2016.01539 27761130 PMC5050229

[B90] Organisation for Economic Co-operation and Development (OECD) (2015). “Biosafety and the environmental uses of micro-organisms,” in Conference proceedings. Paris, France: OECD Publishing. 10.1787/9789264213562-en

[B91] Osman A. I. Fang B. Zhang Y. Liu Y. Yu J. Farghali M. (2024). Life cycle assessment and techno-economic analysis of sustainable bioenergy production: a review. Environ. Chem. Lett. 22, 1115–1154. 10.1007/s10311-023-01590-3

[B92] Parsons S. Chuck C. J. McManus M. C. (2018). Microbial lipids: progress in life cycle assessment (LCA) and future outlook of heterotrophic algae and yeast-derived oils. J. Clean. Prod. 172, 661–672. 10.1016/j.jclepro.2017.10.161

[B93] Patel A. R. Patel G. Srivastava A. Banerjee S. (2023). A review on traditional and modern methods for the synthesis of aromatic azo compounds. Curr. Org. Chem. 27, 1611–1628. 10.2174/0113852728245448231011103950

[B94] Permpornsakul P. Prasongsuk S. Lotrakul P. Eveleigh D. E. Kobayashi D. Y. Imai T. (2016). Biological treatment of reactive black 5 by resupinate white-rot fungus *Phanerochaete sordida* PBU 0057. Pol. J. Environ. Stud. 25, 1205–1212. 10.15244/pjoes/61625

[B95] Ramamurthy K. Priya P. S. Murugan R. Arockiaraj J. (2024). Hues of risk: investigating genotoxicity and environmental impacts of azo textile dyes. Environ. Sci. Pollut. Res. 31, 33190–33211. 10.1007/s11356-023-29542-2 38676865

[B96] Rane A. Joshi S. J. (2021). Biodecolorization and biodegradation of dyes: a review. Open Biotechnol. J. 15, 1–10. 10.2174/1874070702115010001

[B97] Rath S. Pradhan D. Du H. Mohapatra S. Thatoi H. (2024). Transforming lignin into value-added products: perspectives on lignin chemistry, lignin-based biocomposites, and pathways for augmenting ligninolytic enzyme production. Adv. Compos. Hybrid. Mater. 7, 27. 10.1007/s42114-023-00703-4

[B98] Robles-Iglesias R. Naveira-Pazos C. Fernández-Blanco C. Veiga M. C. Kennes C. (2023). Factors affecting optimization and scale-up of lipid accumulation in oleaginous yeasts for sustainable biofuel production. Renew. Sustain. Energy Rev. 171, 113043. 10.1016/j.rser.2022.113043

[B99] Rubinstein C. Levitus G. Vicien C. Modena N. A. Ruzal S. Vesprini F. (2025). Genetically modified microorganisms for agricultural use: an opportunity for the advancement of risk assessment criteria in Argentina. Front. Bioeng. Biotechnol. 13, 1612226. 10.3389/fbioe.2025.1612226 40585850 PMC12202534

[B100] Ruscasso F. Cavello I. Curutchet G. Cavalitto S. (2022). Antarctic yeasts: potential use in a biological treatment of textile azo dyes. Bioresour. Bioproc. 9, 18. 10.1186/s40643-022-00472-1 38647816 PMC10991636

[B101] Sae-Ngae S. Cheirsilp B. Louhasakul Y. Suksaroj T. T. Intharapat P. (2020). Techno-economic analysis and environmental impact of biovalorization of agro-industrial wastes for biodiesel feedstocks by oleaginous yeasts. Sustain. Environ. Res. 30, 11. 10.1186/s42834-020-00063-7

[B102] Saini R. Choudhary K. (2025). “Toxic potential of azo dyes: a broader understanding,” in Hazardous chemicals. Amsterdam, Netherlands: Academic Press (Elsevier), 469–481.

[B103] Salgado J. F. M. Hervé V. Vera M. A. Tokuda G. Brune A. (2024). Unveiling lignocellulolytic potential: a genomic exploration of bacterial lineages within the termite gut. Microbiome 12, 201. 10.1186/s40168-024-01866-x 39407345 PMC11481507

[B104] Sankhla V. Mathew D. Jawalekar S. Singh S. P. Sharma S. Bhatia N. (2025). Urinary bladder cancer with dye exposure in textile industry workers in Pali district, Rajasthan. J. Neonatal Surg. 14, 4s–1070. 10.52783/jns.v14.1914

[B105] Saravanan A. Kumar P. S. Vo D. V. N. Jeevanantham S. Karishma S. Yaashikaa P. R. (2021). A review on catalytic-enzyme degradation of toxic environmental pollutants: microbial enzymes. J. Hazard. Mater. 419, 126451. 10.1016/j.jhazmat.2021.126451 34174628

[B106] Sartaj K. Prasad R. Matsakas L. Patel A. (2023). Transforming recalcitrant wastes into biodiesel by oleaginous yeast: an insight into the metabolic pathways and multi-omics landscape. Chem. Eng. J. 474, 145625. 10.1016/j.cej.2023.145625

[B107] Scharf M. E. (2020). Challenges and physiological implications of wood feeding in termites. Curr. Opin. Insect Sci. 41, 79–85. 10.1016/j.cois.2020.08.002 32823202

[B108] Schwarz M. Tokuda G. Osaki H. Mikaelyan A. (2023). Reevaluating symbiotic digestion in cockroaches: unveiling the Hindgut’s contribution to digestion in wood-feeding Panesthiinae (Blaberidae). Insects 14, 768. 10.3390/insects14090768 37754736 PMC10531843

[B109] Shao Q. Yan S. Sun X. Chen H. Lu Y. Li S. (2025). Applications of yeasts in heavy metal remediation. Fermentation 11, 236. 10.3390/fermentation11050236

[B110] Sharma K. D. Jain S. (2020). Municipal solid waste generation, composition, and management: the global scenario. Soc. Responsib. J. 16 (6), 917–948. 10.1108/SRJ-10-2018-0294

[B111] Sharma P. Saha S. Chatterjee S. Mandal A. H. Ghosh S. Saha N. C. (2025). Toxicological and physiological impact and bioremediation strategies for polycyclic aromatic hydrocarbons (PAHs). Chem. Ecol. 41, 1–24. 10.1080/02757540.2025.2490035

[B112] Show B. K. Banerjee S. Banerjee A. Ghosh Thakur R. Hazra A. K. Mandal N. C. (2022). Insect gut bacteria: a promising tool for enhanced biogas production. Rev. Environ. Sci. Bio/Technol. 21, 1–25. 10.1007/s11157-021-09615-y

[B113] Silva J. D. M. E. Martins L. H. D. S. Moreira D. K. T. Silva L. D. P. Barbosa P. D. P. M. Komesu A. (2023). Microbial lipid-based biorefinery concepts: a review of status and prospects. Foods 12 (10), 2074. 10.3390/foods12102074 37238892 PMC10217607

[B114] Singh A. K. Fernandez-Lafuente R. Schmidt J. E. Boczkaj G. Bilal M. (2024). Biocatalytic functionalities of lignin peroxidase-based systems in lignin depolymerization and pollutant removal from environmental matrices. Curr. Pollut. Rep. 10, 345–361. 10.1007/s40726-024-00263-9

[B115] Sitepu I. R. Garay L. A. Sestric R. Levin D. Block D. E. German J. B. (2014). Oleaginous yeasts for biodiesel: current and future trends in biology and production. Biotechnol. Adv. 32, 1336–1360. 10.1016/j.biotechadv.2014.08.003 25172033

[B116] Siwina S. Leesing R. (2021). Bioconversion of durian (*Durio zibethinus* Murr.) peel hydrolysate into biodiesel by newly isolated oleaginous yeast Rhodotorula mucilaginosa KKUSY14. Renew. Energy 163, 237–245. 10.1016/j.renene.2020.08.084

[B117] Song L. Shao Y. Ning S. Tan L. (2017). Performance of a newly isolated salt-tolerant yeast strain *Pichia occidentalis* G1 for degrading and detoxifying azo dyes. Bioresour. Technol. 233, 21–29. 10.1016/j.biortech.2017.02.053 28258992

[B118] Sun J. Ding S.-Y. Peterson J. D. (2013). Biological conversion of biomass for fuels and chemicals: explorations from natural utilization systems (Cambridge, United Kingdom: Royal Society of Chemistry). 10.1039/9781849734738

[B119] Szczepańska P. Hapeta P. Lazar Z. (2022). Advances in production of high-value lipids by oleaginous yeasts. Crit. Rev. Biotechnol. 42, 1–22. 10.1080/07388551.2021.1873259 34000935

[B120] Tabañag I. D. F. Chu I. M. Wei Y.-H. Tsai S.-L. (2018). The role of yeast-surface-display techniques in creating biocatalysts for consolidated bioprocessing. Catalysts 8, 94. 10.3390/catal8030094

[B121] Tan L. He M. Song L. Fu X. Shi S. (2016). Aerobic decolorization, degradation, and detoxification of azo dyes by a newly isolated salt-tolerant yeast *Scheffersomyces spartinae* TLHS-SF1. Bioresour. Technol. 203, 287–294. 10.1016/j.biortech.2015.12.055 26744802

[B122] Tan L. Xu B. Hao J. Wang J. Shao Y. Mu G. (2019). Biodegradation and detoxification of azo dyes by a newly isolated halotolerant yeast *Candida tropicalis* SYF-1. Environ. Eng. Sci. 36, 999–1010. 10.1089/ees.2019.0200

[B123] Thak E. J. Yoo S. J. Moon H. Y. Kang H. A. (2020). Yeast synthetic biology for designed cell factories producing secretory recombinant proteins. FEMS Yeast Res. 20, foaa009. 10.1093/femsyr/foaa009 32009173

[B124] Tiwari S. Avchar R. Arora R. Lanjekar V. Dhakephalkar P. K. Dagar S. S. (2020). Xylanolytic and ethanologenic potential of gut-associated yeasts from different species of termites in India. Mycobiology 48, 501–511. 10.1080/12298093.2020.1832677 33312017 PMC7717550

[B125] Tripathi P. Alshahrani S. Alhazmi H. A. Tripathi R. Siddiqui A. H. Ahsan W. (2020). *In vivo* assessment of genotoxic potential of brown shammah (smokeless tobacco) in bone marrow cells of mice. Saudi Pharm. J. 28, 480–486. 10.1016/j.jsps.2020.02.006 32273808 PMC7132595

[B126] Velasquez-Orta S. Mohiuddin O. (2023). “The utilization of yeast for industrial wastewater treatment,” in Advances in yeast biotechnology for biofuels and sustainability (Amsterdam, Netherlands: Elsevier), 353–370. 10.1016/B978-0-323-91173-9.00017-8

[B127] Wang X. Yao B. Su X. (2018). Linking enzymatic oxidative degradation of lignin to organics detoxification. Int. J. Mol. Sci. 19, 3373. 10.3390/ijms19113373 30373305 PMC6274955

[B128] Wang Y. Xu B. Ning S. Shi S. Tan L. (2021). Magnetically stimulated azo dye biodegradation by a newly isolated osmo-tolerant *Candida tropicalis* A1 and its transcriptomic responses. Ecotoxicol. Environ. Saf. 209, 111791. 10.1016/j.ecoenv.2021.111791 33360211

[B129] Wen H. Xiong K. Yang H. Zhang P. Wang X. (2022). Dynamic mechanism of the microbiota of high-salinity organic wastewater with salt-tolerant yeast and its application. J. Environ. Chem. Eng. 10, 107377. 10.1016/j.jece.2022.107377

[B130] Xie R. Danso B. Sun J. Al-Zahrani M. Dar M. A. Al-Tohamy R. (2024). Biorefinery and bioremediation strategies for efficient management of recalcitrant pollutants using termites as an obscure yet promising source of bacterial gut symbionts: a review. Insects 15, 908. 10.3390/insects15110908 39590507 PMC11594812

[B131] Yan L. Huertas-Alonso A. J. Liu H. Dai L. Si C. Sipponen M. H. (2025). Lignin polymerization: towards high-performance materials. Chem. Soc. Rev. 54, 6634–6651. 10.1039/D4CS00789A 40491312 PMC12150016

[B132] Yang M. Zheng S. (2014). Pollutant removal-oriented yeast biomass production from high-organic-strength industrial wastewater: a review. Biomass Bioenergy 64, 356–362. 10.1016/j.biombioe.2014.02.017

[B133] Ye M. Ye Y. Du Z. Chen G. (2021). Cell-surface engineering of yeasts for whole-cell biocatalysts. Bioprocess Biosyst. Eng. 44, 1003–1019. 10.1007/s00449-020-02446-3 33389168

